# A scoping review of novel spinal cord stimulation modes for complex regional pain syndrome

**DOI:** 10.1080/24740527.2019.1574536

**Published:** 2019-03-05

**Authors:** Yasmine Hoydonckx, Matteo Costanzi, Anuj Bhatia

**Affiliations:** a Department of Anesthesia and Pain Medicine, University of Toronto, Toronto Western Hospital, Toronto, Ontario, Canada; b Department of Anesthesiology and Intensive Care Medicine, Università Cattolica del Sacro Cuore, Fondazione Policlinico Universitario Agostino Gemelli, Rome, Italy; c Institute of Health Policy Management and Evaluation, University of Toronto, Toronto, Ontario, Canada

**Keywords:** spinal cord stimulation, paresthesia-free stimulation, high-frequency stimulation, burst stimulation, high-density stimulation, CRPS

## Abstract

**Background**: Paresthesia-based spinal cord stimulation (PB-SCS) is used for the treatment of complex regional pain syndrome (CRPS), but many patients are refractory to PB-SCS or experience attenuation of analgesic effect over time due to tolerance. Novel SCS modes including high-frequency, Burst^TM^, and high-density (HD^TM^) stimulation were introduced recently and this systematic review was conducted to summarize the evidence on their role for CRPS.

**Materials and Methods**: We searched MEDLINE and other databases (up to September 21, 2017) for studies including adults with refractory CRPS treated by paresthesia-free SCS (PF-SCS) modes compared to placebo, conventional medical treatment, or PB-SCS. We determined the posttreatment intensity of pain (up to 24 months after intervention), changes in CRPS-associated symptoms, and associated domains. Sustainability and adverse effects were also assessed.

**Results**: We identified 13 studies (seven case series, five conference abstracts, one randomized controlled trial) including 62 patients with upper or lower limb CRPS. Eleven papers reported on outcomes of high-frequency stimulation at 10 kHz (HF-10) and other high frequencies, two papers were on Burst, and one paper was on HD. In 59 patients, pain intensity with novel SCS modes was reduced by 30% to 100% with a corresponding reduction in analgesic medications. Novel SCS modes also attenuated CRPS-associated symptoms and six papers reported significant improvement of quality of life.

**Conclusions**: Novel SCS modes have the potential to provide analgesia in patients with CRPS. However, the low quality of available evidence necessitates definitive and prospective comparative effectiveness studies to establish the role of these modes in CRPS.

## RÉSUMÉ

**Contexte**: La stimulation médullaire par induction de paresthésies est utilisée pour le traitement du syndrome douloureux régional complexe (SDRC), mais de nombreux patients y sont réfractaires ou ressentent une atténuation de l’effet analgésique avec le temps dû à la tolérance. De nouvelles modalités de stimulation médullaire, dont la stimulation à haute fréquence, la stimulation par rafales (Burst^TM^) et la stimulation à haute densité (HD^TM^), ont récemment facommentcé à être utilisés. Cette revue systématique a été menée afin de résumer les données probantes portant sur leur rôle dans le SDRC.

**Matériel et méthodes**: Nous avons fait des recherches dans MEDLINE et d’autres bases de données (jusqu’au 21 septembre 2017) pour trouver des études portant sur des adultes souffrant d’un SDRC réfractaire traité par stimulation médullaire sans parasthésie comparativement à un placebo, à un traitement médical conventionnel ou à une stimulation médullaire par induction de paresthésies. Nous avons déterminé l’intensité post-traitement de la douleur (jusqu’à 24 mois après l’intervention), ainsi que les changements dans les symptômes associés au SDRC et dans des domaines connexes. La pérennité et les effets défavorables ont aussi été évalués.

**Résultats**: Nous avons répertorié 13 études (7 séries de cas, 5 résumés de conférence, un essai contrôlé randomisé) auxquelles ont participé 62 patients souffrant d’un SDRC aux membres supérieurs ou inférieurs. Onze articles portaient sur les résultats de la HF-10 et d’autres hautes fréquences, deux articles portaient sur la stimulation *Burst*^TM^ et un article portait sur la HD^TM^. Chez 50 patients, les modalités novatrices de stimulation médullaire ont donné lieu à une diminution de l’intensité de la douleur allant de 30 % à 100 %, accompagnée d’une réduction similaire des médications analgésiques. Les nouveaux modes de stimulation médullaire atténuaient également les symptômes associés au SDRC. De plus, six articles faisaient état d’une amélioration considérable de la qualité de vie.

**Conclusions**: Les nouveaux modes de stimulation médullaire peuvent avoir un effet analgésique pour les patients souffrant d’un SDRC. Toutefois, la qualité médiocre des données probantes disponibles rend nécessaire la conduite d’études comparatives et prospectives sur leur efficacité des afin d’établir leur rôle dans le SDRC.

## Introduction

Complex regional pain syndrome (CRPS) is characterized by spontaneous and or evoked regional pain that is seemingly disproportionate in time or degree to the usual course of any known trauma or other lesion. The pain is regional but may not (CRPS type I) or may be (CRPS type II) in the distribution of a specific nerve territory or dermatome. CRPS usually has a predominance of abnormal sensory, motor, sudomotor, vasomotor, and trophic findings and it shows variable progression over time.^1^ Patients often develop severe functional disability, depression, and social isolation as a consequence of CRPS. The incidence of this syndrome is 5.46–26.2 per 100 000 person-years with a prevalence of 20.57 cases per 100 000 person-years.^2^ More women than men seem to be affected and the upper extremity is the most commonly involved body part.^3^ Though identifying this syndrome accurately can be a challenge, the criteria to diagnose CRPS were refined in 2007 to improve the specificity and reduce sensitivity.^4^ Several mechanisms seem to play a role in the development of CRPS, including inflammation, vasomotor dysfunction, central sensitization, neuroplasticity, and autoimmune involvement.^5^

Though a variety of treatment modalities have been proposed for treatment of CRPS, a significant number of patients experience unsatisfactory pain relief and/or have adverse effects from existing pharmacological or neuromodulatory interventions. Pharmacological therapies include nonsteroidal anti-inflammatory drugs, corticosteroids, anticonvulsants, antidepressants, dimethylsulfoxide, N-acetylcysteine, transdermal patches (lidocaine, capsaicin), intravenous infusions of ketamine, and vasodilators.^1,6,7^ Physical therapies should be the mainstay of treatment and these include desensitization, restoration of range of motion, and strength of muscles in the affected extremity. Interventional approaches include neural plexus and sympathetic chain blocks. However, failure to relieve pain and resolve associated symptoms of CRPS is common with these pharmacological and physical therapies.

Neuromodulation approaches including spinal cord, dorsal root ganglia, and peripheral nerve stimulation have been used for chronic pain syndromes with traditionally neuropathic but more recently nociceptive pathologies, including failed back surgery syndrome, CRPS, and other neuropathic pain syndromes.^8,9^ Paresthesia-based spinal cord stimulation (PB-SCS), with stimulating frequencies between 30 and 60 Hz that confer a tingling sensation, has been extensively used to treat CRPS over the last 2 decades but it suffers from limitations including attenuation of benefit with time and painful or unwanted paresthesias. The recent increase in availability and use of novel SCS modes promises to address these shortcomings.

There are three new stimulation modes currently in use: high-frequency stimulation at 10 kHz (HF-10), high-density (HD^TM^), and Burst^TM^ stimulation. It has been proposed these modes share the property of delivering higher amounts of electric charge to the spinal cord, or perhaps the same amount of charge with a higher efficacy, with frequencies in the range of 500 Hz to 10 kHz and stimulation amplitudes that are below the perception threshold.^10^ These “higher” stimulation frequencies potentially modulate (i.e., suppress) action potentials that contribute to painful sensations or sympathetic dysregulation but come with the advantage of being paresthesia free.^11^ It has been suggested that in patients with failure of conventional low-frequency PB-SCS to achieve analgesia, at the time of either SCS trial or loss of benefit with passage of time despite initial success, increasing the stimulation frequency or changing the stimulation pattern to deliver one of the novel SCS modes could confer analgesic benefit despite the absence of paresthesias.^12^

Though the three novel SCS modes share the feature of not having paresthesias, there are some differences among these three modes due to unique combinations of frequency, subperception threshold amplitude, and pulse width. Clinically used high-frequency SCS is usually at a stimulation frequency of 10 kHz applied via bipolar stimulation but any stimulating frequency above 1 kHz has been considered as high.^11^ Burst stimulation consists of multiple burst complexes with an overall frequency of 40 Hz. One burst complex contains five spikes at 500 Hz and pulse width of 1000 ms, delivered with a 40-Hz frequency and charge-balanced at the end of each burst.^10^ The key features of HD SCS are an increase in stimulation frequency in the range of 500 to 1200 Hz without decreasing the pulse width from the setting used in PB-SCS, thereby increasing the “pulse density” (i.e., the percentage active stimulation during a pulse cycle).^10,13^

It is not surprising that there has been an increasing interest in the use of PF-SCS in patients with CRPS, a cohort that lacks access to effective analgesic options. There are now a number of recent publications and conference presentations on this topic. The objectives of this scoping review were to evaluate the analgesic impact and adverse effects of PF-SCS on patients with CRPS who had received these modes either as a primary neuromodulation treatment or when it was introduced following failure of PB-SCS.

## Materials and methods

### Data sources and search

This scoping review was conducted according to the recommendations of the Cochrane Collaboration and it is reported as per the Preferred Reporting Items for Systematic Reviews and Meta-Analysis (PRISMA) guidelines (Appendix 1).^14,15^ This review was registered with PROSPERO (an international prospective register of systematic reviews; #CRD42017077931).

We conducted comprehensive, serial searches of the literature from inception to September 21, 2017, with the assistance of a medical information specialist. The following databases were searched: EMBASE, 1947 onward; MEDLINE, 1946 onward; MEDLINE In-Process and Other Non-Indexed Citations (all using the OvidSP Platform); and Cochrane Database of Systematic Reviews. PROSPERO and Cochrane Central Register of Controlled Trials were included to identify reviews or trials that may have been published but missed during the initial search on MEDLINE and EMBASE. We also searched Google Scholar (first 200 search results were reviewed) to complement search results from the aforementioned databases with the objective of accessing all content relevant to the topic. Proceedings of the major annual meetings of anesthesiology and pain societies (International Neuromodulation Society, North American Neuromodulation Society, American Society of Anesthesiologists, European Society of Anaesthesiology, International Association for the Study of Pain [IASP], American Society of Regional Anesthesia and Pain Medicine, European Society of Regional Anesthesia and Pain Therapy, and World Institute of Pain) in the last 2 years were also searched. We also searched for trials in the metaregister of controlled trials (www.clinicaltrials.gov). Papers in all languages were included when it was possible to translate them into English. Examples of specific search terms used include “spinal cord stimulation,” “neuromodulation,” “burst stimulation or neuromodulation,” “paresthesia-free stimulation,” “high frequency stimulation or neuromodulation,” “high density stimulation or neuromodulation,” “stimulation frequency above 500 kHz,” “complex regional pain syndrome (CRPS),” “algodystrophy,” “Südeck’s atrophy,” and “reflex sympathetic dystrophy.” Boolean operators (and, or) were used to combine search terms. The scope of studies included randomized controlled trials, systematic reviews, observational or cohort studies, case–control studies, case series/reports, and conference abstracts.

### Inclusion criteria and study selection

We prespecified eligibility criteria using the population, intervention, comparator, and outcomes model as follows:

- Population: Studies included in the clinical analysis focused on adult patients (at least 18 years of age), refractive to conventional medical management and or conventional PB-SCS.
- Intervention: The interventions of interest were the novel SCS modes, including Burst, high-frequency (HF-10), and HD stimulation.
- Comparator: Comparators included placebo treatment or conventional medical management or conventional PB-SCS.
- Outcome: Our primary outcome was change in intensity of pain assessed on a numeric rating scale (NRS) or visual analogue scale (VAS) at 1 to 12 months after initiation of the intervention. Secondary outcomes of interest included change in CRPS-associated vasomotor, sudomotor, and trophic symptoms and impact of SCS on pain-associated domains (functional outcome, psychological outcome, quality of life, return to work, patient satisfaction, or global impression of change). Sustainability of analgesic benefit and adverse effects were also noted.

Titles, abstracts, and, when required, full text of the papers identified from the initial search were examined for relevance as per the inclusion criteria for this review. Only studies that met the abovementioned criteria were included for data extraction. We completed the search by reviewing the bibliographies of every selected article to look for possible additional articles that were not identified by the initial search.

### Data extraction

The reference data, populations, and outcomes were extracted from the articles into prespecified tables using a standardized data extraction form. The data collection form was pilot-tested before its use. We extracted information on studies’ source (study ID and reviewer ID), number of patients, type of study, patient characteristics, details of intervention, comparator group where reported, previous treatments, follow-up time points, outcomes, and adverse effects of the intervention. Dichotomous outcomes were extracted where indicated. For continuous data, extraction of means (or medians) and standard deviations (or interquartile ranges or ranges) was performed. We also contacted authors of studies included in our scoping review when we needed more information about their analysis or reported results.

Data were independently extracted by two reviewers (Y.H. and M.C.) and any conflicts were resolved by consensus and inputs from the senior author (A.B.).

### Risk of bias

Two review authors (Y.H. and A.B.) independently assessed the risk of bias for randomized controlled trials using the Cochrane Collaboration’s instrument for assessing the risk of bias. Any disagreement was resolved through discussion or, if necessary, arbitration by the senior author (A.B.). The risk of bias instrument assesses the following domains: generation of the allocation sequence, allocation concealment, blinding of investigators and participants, blinding of outcome assessors, incomplete outcome data, selective outcome reporting, and other sources of bias that have less empirical evidence of bias but together may be considered important (unequal distribution of prognostic factors, industry funding, industry authorship, trial stopped early). Each item was classified as low, unclear, or high risk of bias. A decision to classify “overall bias” as low, unclear, or high was made by the reviewers using the following method:

- High: Any trial with a high risk of bias listed on three or more domains.
- Unclear: Any trial with a high risk of bias listed on more than one domain but less than three domains.
- Low: Any trial with a high risk of bias on no domain or one domain and with no significant methodologic concerns that may have affected the study results.

The quality of the case series was assessed using the quality appraisal tool developed by Moga et al.^16^ This tool has 18 items with three response choices to each question: Yes, unclear/partially reported, or no.

### Data synthesis

Our synthesis of clinical studies was anticipated to focus on results from randomized controlled trials followed by prospective trials or case series or reports. Limited scope for meta-analysis was anticipated due to the limited number, clinical heterogeneity, methodological diversity, and paucity of clinical trials.

## Results

### Search results

Our initial search revealed a total of 2099 records. We found another three articles while perusing references from articles that were short-listed and 15 records were found in the clinical trial registries. Details of the search strategy for high-frequency SCS for treatment of CRPS are provided in Appendix 2. Similar methodology was followed for searches for role of Burst and HD SCS in CRPS.

**Figure 1.**
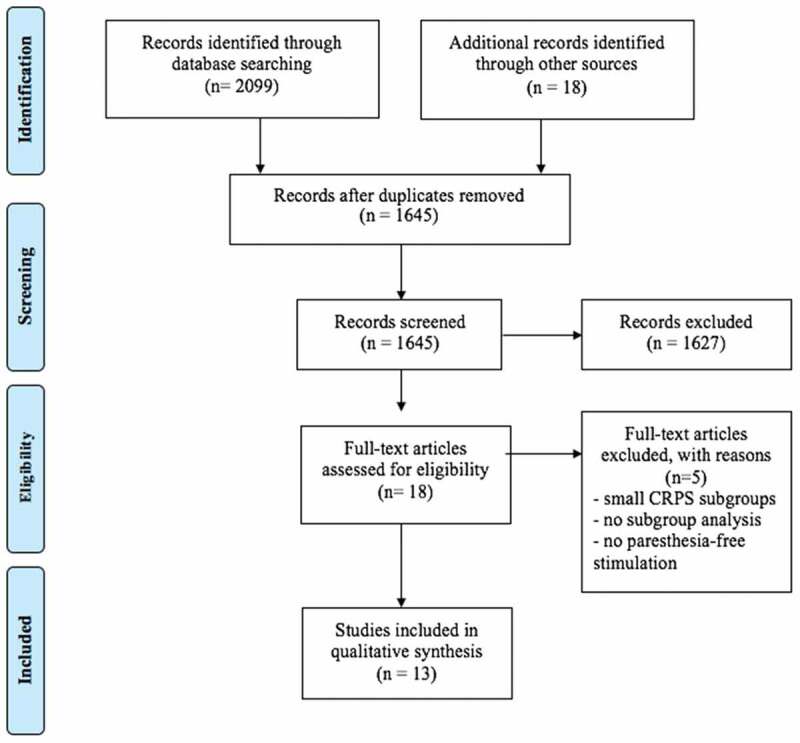
PRISMA flowchart for the systematic review.

After removal of duplicates, 1645 records remained and were title and abstract screened as per our population, intervention, comparator, and outcomes criteria specified in the Methods section; 1627 records were excluded based on title and abstract screening because of lack of relevance or absence of information about our primary and secondary outcomes. Full text of the remaining 18 articles was assessed for eligibility. Another five articles were excluded because of data on only a small subgroup of patients with CRPS, due to lack of subgroup analysis for CRPS, or because no patients received stimulation with any of the novel SCS modes. Thirteen studies fulfilled our inclusion criteria (Figure 1) and these included seven case series or reports,^12,13,17–21^ five conference abstracts,^22–26^ and one randomized controlled trial (RCT^27^) (Tables 1 and 2).

**Table 1.** Characteristics of studies included in the systematic review: Participants, interventions, and comparators.

Study, number of patients, type of study	Participants and pain syndromes	Intervention: Details of paresthesia-free SCS	Comparator group	SCS lead type and position	Therapies tried prior to paresthesia-free SCS
**Case reports/series**					
Al-Kaisy et al.^12^ 2015 (*n* = 15) CS (R)	Age: 46 ± 12 years M/F: 5/6 Pre-intervention: NRS pain: 8.2 ± 1.7 BPI 57.6 ± 9.4 painDETECT 29 ± 8 PCS 33 ± 11 CRPS (6 patients): UL (3), LL (3) (IASP Budapest criteria used) Duration of CRPS: NP	Type: HF-10 F: 10 kHz PW: NP A: 0.5−3.5 mA Intraoperative testing with PB-SCS to ensure >80% overlap of stimulation with patients’ pain locations	—	Trial type: Percutaneous or tunneled SCS leads: 1–2 octopolar cylindrical leads Lead level: UL: C2–C7 LL: T8–T12	CMM: TCA, gabapentinoids, opioids Topical treatments Physical therapy TENS CBT
Crapanzano et al.^17^ 2017 (*n* = 1) CR (R)	Age: 53 years F CRPS knee and thigh (spread to bilateral arms/shoulders over time) NRS pain: NP Duration of CRPS: 7 years	Type: HF-10 F: 10 kHz PW: NP A: NP	—	NP	CMM: Opioids, NSAIDs, TCA, SNRI, gabapentinoids Topical treatments Physical therapy LSB
Kriek et al.^18^ 2015 (*n* = 1) CR (R)	Age: 65 years F CRPS: UL NRS pain: 8 Duration: 16 years	Type: Burst F: 500/40 Hz PW: 1000 ms A: 0.225	Failure of PB-SCS after 4 years of good analgesia F: 60 Hz PW: 387 ms A: 5.8V	Trial type: NP SCS lead: 1 octopolar cylindrical lead Lead level: NP	CMM: NSAIDs, TCA, pregabalin sympathetic blockade TENS Physical therapy
Reddy et al.^19^ 2016 (*n* = 2) CS (P)	Age: 56.5 years M/F: 0/2 CRPS lower extremity NRS pain: 8 (6–10)^a^ Duration: 1.6 (0.4–2.9) years	Type: HF-10 F: 400 Hz–10 kHz PW: NP A: 0.5–10 V	—	Trial type: NP SCS lead: NP Lead level: T8–T11	NP
Smith et al.^20^ 2015 (*n* = 1) CR (R)	Age: 70 years F CRPS back and LL NRS pain: 6 Duration: 20 years (?)	Type: HF F: 1.15 kHz PW: 120 ms A: 5.0 V	Failure of PB-SCS after 6 months of good analgesia F: 40 Hz PW: 330 ms A: 3.5 V	Trial type: SCS in situ SCS lead: paddle Lead level: top at caudal aspect of T8	CMM Physical therapy Epidural injections RF lesioning
Tate^21^ 2017 (*n* = 1) CR (R)	Age: 56 years F CRPS right LL + phantom limb pain NRS pain: NP Duration: 12 years	Type: HF-10 F: 10 kHz PW: NP A: NP	Failed trial of PB-SCS due to intolerable paresthesias	Trial type: NP SCS lead: 2 leads (NP) Lead level: T8–T11	CMM Injections (NP) Conventional SCS trial: failed LSB (RF) Nerve blocks
Wille et al.^13^ 2017 (*n* = 3) CS (R)	Age: 49 years M/F: NP CRPS: details NP NRS pain: 7.9 Duration: NP	Type: HD F: 130–1000 Hz (mean 409 Hz) PW: 150–1000 ms A: 2.4 V (subperception)	Failure of PB-SCS over time and unwanted paresthesias F: 30–60 Hz PW: 284 ms A: 3.3 V	Trial type: NP SCS lead: NP Lead level: as close as possible to T9–T10 disc interspace	
**Abstracts**					
Amirdelfan et al.^22^ 2017 (*n* = 6) CS (R)	Age: NP M/F: NP CRPS UL (1) CRPS LL (5 – all except 1 had bilateral CRPS) NRS pain: 7.8 (mean) Duration: NP	Type: HF-10 F: 10 kHz PW: 30 ms A: individualized	—	Trial type: NP SCS leads: 1–2 octopolar cylindrical leads Lead level: UL: C2–C6 LL: T8–T11	NP
Santarelli et al.^23^ 2016 (*n* = 7) CS (P)	Age: 39–75 years M/F: 5/2 CRPS: UL (2) LL (5) (IASP Budapest criteria used) NRS pain: 6–10 Duration: 9 months–4 years	Type: HF-10 F: 10 kHz PW: NP A: NP	—	Trial type: NP SCS leads: 1–2 octopolar cylindrical leads Lead level: UL: C2–C6 LL: T8–T11	NP
Smet and Van Buyten^24^ 2016 (*n* = 1) CR (R)	Age: 59 years F CRPS: UL NRS pain: 7–10 Duration: > 1 year	Type: HF-10 F: 10 kHz PW: NP A: NP	Initial positive trial of PB-SCS but loss of effect	Trial type: NP SCS leads: 2 octopolar leads Lead level: C2–C5	CMM
Gulve et al.^25^ 2015 (*n* = 1) CR (R)	Age: 63 years M CRPS: UL NRS pain: 7–10 Duration: 7 years	Type: HF-10 F: 10 kHz PW: NP A: NP	—	Trial type: percutaneous SCS lead: 1 octopolar lead Lead level: tip at C1/C2	CMM: opioids, TCA, gabapentinoid Physiotherapy
Wohak^26^ 2013 (*n* = 3) CS (R)	Age: 37–48 years M/F: 2/1 CRPS: UL NRS pain: NP Duration: NP	Type: HF-10 F: 10 kHz PW: NP A: NP	PB-SCS with initial good result in one patient but failure after dislocation	Trial type: NP SCS lead: 1–2 octopolar leads Level: cervical spine	CMM: opioid, pregabalin, NSAIDs
**Randomized controlled trial**					
Kriek et al.^27^ 2017 (*n* = 29) RCT	Age: 42.6 ± 12.8 years M/F: 4/25 CRPS: UL (11), LL (18) VAS: 72.7 ± 2.6 mm Duration: 3 (1–5)	Type: Three groups: 500 Hz, 1200 Hz, Burst	Type: PB-SCS (40 Hz) and placebo groups	Trial type: NP SCS lead: 1 octopolar lead Level: NP but “optimal positioning with overlapping paresthesia in the painful area”	NP

SCS = spinal cord stimulation; CS (R/P) = case series (retrospective/prospective); M/F = male/female; NRS = numerical rating scale; BPI = Brief Pain Inventory; PCS = Pain Catastrophizing Scale; CRPS = complex regional pain syndrome; UL = upper limb; LL = lower limb; IASP = International Association for the Study of Pain; NP = not provided; HF-10 = high-frequency stimulation at 10 kHz; F = frequency; PW = pulse width; A = amplitude; PB-SCS = paresthesia-based spinal cord stimulation; CMM = conventional (non-SCS) medical management; TENS = transcutaneous electrical nerve stimulation; CBT = cognitive behavioral therapy; CR = case report; NSAIDs = nonsteroidal anti-inflammatory drugs; TCA = tricyclic antidepressants; SNRI = serotonin and norepinephrine reuptake inhibitors; LSB = lumbar sympathetic plexus block; HF = high-frequency stimulation; RF = radio frequency; HD = high-density stimulation; RCT = randomized controlled trial; VAS = visual analog scale.

**Table 2.** Characteristics of studies included in the systematic review: Outcomes including analgesic benefits and adverse effects.

Study, year, number of patients, type of study	Newer SCS mode	Follow-up time points after implantation	Outcomes assessed	Important results	Adverse effects of paresthesia-free SCS	Comments
**Case reports/series**						
Al-Kaisy et al.^12^ 2015 (*n* = 15) CS (R)	HF-10	1, 3, and 6 months	NRS pain BPI PCS EQ-5D painDETECT	At 6 months: NRS pain reduced by 59% BPI reduced by 45% PCS reduced by 75% EQ-5D increased by 101% Excellent or good satisfaction for 10/11 patients	No adverse effects related to paresthesia-free SCS	11/15 had successful trials but 3 of the 4 patients who had no benefit had CRPS of the foot; all 3 patients with CRPS of the hand responded
Kriek et al.^18^ 2015(*n* = 1) CR (R)	Burst	24 months	NRS pain Medication intake Vasomotor and sudomotor symptoms ROM	NRS pain decreased by 75% Lower medication intake No worsening in vasomotor and sudomotor symptoms or ROM	—	Burst stimulation provides analgesia when tolerance develops to conventional SCS
Reddy et al.^19^ 2016 (*n* = 2) CS (P)	HF-10	0.5 days	VAS pain Discomfort Feedback from patients	NRS pain reduced by 30%–50% Patients preference: HF		0.5-day trial of HF-SCS during weeklong conventional trial; possibility of carryover effects
Smith et al.^20^ 2015 (*n* = 1) CR (R)	HF (1.15 kHz)	6 months	NRS pain QoL Medication intake Quality of sleep	NRS pain reduced by 50% Reduction of medication intake	—	Paresthesia-free (high-frequency) SCS mode used by patient to avoid intolerable paresthesias
Tate^21^ 2017(*n* = 1) CR (R)	HF-10	4 months	NRS pain QoL Vasomotor and sudomotor symptoms Anxiety ROM Medication intake	100% pain relief with resolution of phantom limb pain, anxiety, and improved QoL Improvement in vasomotor and sudomotor symptoms ROM: improved gait Medication intake: opiate-free	—	—
Wille et al.^13^ 2017(*n* = 3) CS (R)	HD	1, 6, and 12 months after switch from conventional to HD SCS	NRS pain	Significant and progressive decrease (7.9 to 3.1) in NRS pain scores following implementation of HD mode for 12 months in 2 patients on HD for this duration One patient with CRPS requested reprogramming to conventional SCS due to preference to PB-SCS (although effect of PF-SCS)	—	Possibility of a dose-related response between the amount of energy delivered to the spinal cord and clinical effect
**Abstracts**						
Amirdelfan et al.^22^ 2017 (*n* = 6) CS (R)	HF-10	4 to 15 months	VAS pain Medication usage Physical activity Vasomotor signs	Decrease in VAS pain scores by 83% ± 3.0% and medication usage Improvement in activity and vasomotor signs	—	All 6 patients responded
Santarelli et al.^23^ 2016 (*n* = 7) CS (P)	HF-10	3, 6, 9, and 12 months	VAS pain scores Medication usage Quality of life (SF-36) CES-D scores PGIC Functional status (6-min walking test or hand grip test)	Follow-up ongoing at time of publication of abstract: long-term results not available for every patient At 3 months (*n* = 4): decrease in VAS pain scores (by 25%–100%) and in CES-D scores; improved ROM and PGIC scores At 12 months (*n* = 1): decrease in VAS pain scores to 0 at 12 months and decrease in CES-D scores by 66% with improvement in physical function	—	5 patients had positive trials and 1 patient had direct implant (due to diabetes)
Smet and Van Buyten^24^ 2016 (*n* = 1) CR (R)	HF-10	12 months	NRS pain ROM wrist Medication intake	NRS pain reduced to 0/10 ROM in wrist improved Discontinuation of all analgesic medications	—	—
Gulve et al.^25^ 2015 (*n* = 1) CR (R)	HF-10	18 months	NRS pain EQ-5D score Medications Functional improvement	NRS pain: 80% reduction EQ-5D score increased from 0.2 to 0.8 Discontinuation of all analgesic medications and able to resume pre-injury activities		No postural variation in analgesic effect and no sensory symptoms
Wohak^26^ 2013 (*n* = 3) CS (R)	HF-10	4 to 8 months	NRS pain ROM Medication intake Vasomotor signs	NRS pain and hyperalgesia decreased by 80%–100% ROM: improvement	—	
**Randomized clinical trial**						
Kriek et al.^27^ 2017 (*n* = 29) RCT	Three groups: 500 Hz, 1200 Hz, Burst	At the end of 2 weeks with each of the fove modes At the end of 3 months with the preferred stimulation mode	VAS for pain MPQ GPE Preference of type of stimulation	VAS pain: All PF-SCS settings were equi-analgesic and significantly better with higher GPE scores than placebo VAS pain scores were lower with preferred stimulation 52% of patients preferred nonstandard stimulation	No serious adverse effects	Various reasons for patients preferring particular modes: Amount of pain reduction Lack of paresthesia User-friendliness comfort Recharging time

SCS = spinal cord stimulation; CS (R/P) = case series (retrospective/prospective); HF-10 = high-frequency stimulation at 10 kHz; NRS = numerical rating scale; BPI = Brief Pain Inventory; PCS = Pain Catastrophizing Scale; EQ-5D = EuroQol–five dimensions; CR = case report; ROM = range of motion; HF = high-frequency stimulation; VAS = visual analog scale; QoL = quality of life; HD = high-density stimulation; CRPS = complex regional pain syndrome; PB-SCS = paresthesia-based spinal cord stimulation; PF-SCS = parasthesia-free spinal cord stimulation; SF-36 = Short Form 36; CES-D = Center for Epidemiological Studies–Depression Scale; PGIC = Patient Global Impression of Change; RCT = randomized controlled trial.

### Assessment of quality and risk of bias

The results for assessment of the quality of case series included in this review are listed in Appendix 3. Overall, the quality of the case series was moderate with the number of “yes” responses suggesting adherence to quality metrics (maximum possible score is 18) varying from 10 to 13 in the three studies. The overall risk of bias was deemed to be low for the single RCT in this review.^27^

#### Demographics and pain profiles of study cohort participants

The selected papers included a total of 62 patients with CRPS, of whom the majority were females. The age range of patients was between 30 to 75 years and pain intensity in all patients was severe with NRS of 6/10 (or VAS of 60/100) or higher. A diagnosis of CRPS with details of the affected anatomy was provided in all papers except one.^13^ Use of the IASP Budapest criteria to diagnose CRPS^4^ was stated only in two papers.^12,23^ Twenty-three patients had CRPS in the upper limbs and 39 patients had CRPS in the lower limbs. One publication did not provide details about the location of CRPS but positioning of the SCS leads suggested that patients had CRPS in the lower limbs.^13^ The duration of CRPS prior to interventions ranged from 5 months to 16 years but this information was not provided in four papers.^12,13,22,26^

### Modes of novel SCS

A majority (ten) of these studies involved use of HF-10 SCS therapy,^12,17,19,21–27^ two described use of Burst SCS mode,^18,27^ one described use of HD SCS mode,^13^ and three studies described use of high-frequency stimulation in the 500 to 10 000 Hz range.^19,20,27^ The pulse width of HF-10 SCS waves was provided in only one study^22^ (30 ms) and another study on SCS at 1.15 kHz reported the pulse width as 120 ms.^20^ Of the only two studies on high-frequency SCS up to 10 kHz that reported on the stimulation amplitude, the amplitude range was 0.5–3.5 mA^12^ and 0.5–10 V^19^ and the study with SCS at 1.15 kHz reported the amplitude as 5.0 V.^20^ The two studies on Burst SCS described use of similar parameters: pulse frequency of 500 Hz with a burst frequency of 40 Hz, amplitude of 0.225 mA, and pulse width of 1000 ms.^18,27^ The solitary paper on use of HD SCS in patients with CRPS reported use of a frequency range between 130 and 1000 Hz (median 409 Hz), amplitude of 2.4 V, and pulse width ranging from 150 to 1000 ms.^13^

### Therapeutic modalities used prior to novel SCS modes or as comparators

CRPS is a challenging condition to treat and it is not unusual for a variety of therapeutic modalities to be trialed before an effective option is found. Out of the 62 patients in this review, 15 patients had failed treatment with a multitude of approaches prior to use of PF-SCS. These previous therapies included conventional medical management (first- and second-line medications for treatment of neuropathic pain including tricyclic antidepressants, gabapentinoids, opioids, serotonin, and norepinephrine reuptake inhibitors), peripheral nerve and lumbar sympathetic blocks or radiofrequency lesioning, topical treatments, physiotherapy, transcutaneous electrical nerve stimulation (TENS), cognitive behavioral therapy (CBT), and PB-SCS.^12,17,18,20,21,24–26^

A total of eight patients in four reports were previously treated with conventional PB-SCS with analgesic benefit for up to 4 years.^13,18,20,26^ Though details were not provided, gradual development of tolerance to PB-SCS with increase in intensity of pain after several years of pain relief and/or patients’ dislike of paresthesias (or paresthesias in nonpainful body regions) associated with PB-SCS were the common themes that instigated trials of PF-SCS. Two studies reported failure of PB-SCS during the trial phase.^21,24^ Only one RCT conducted a direct comparison of multiple SCS modes: 500 Hz, 1200 Hz, Burst, conventional, and placebo.^27^ All five modes were programmed in random order during the 10-week crossover period with 2 weeks per setting in this study.

### Technical details of PF-SCS

#### Lead positioning

In the 23 patients with documented CRPS of the upper limb, the stimulating contacts of SCS leads were at the second to seventh cervical vertebral (C2–C7) levels for HF-10,^12,22–26^ Burst,^18,27^ and other high-frequency SCS modes.^27^ In the 39 patients with CRPS of the lower limb, stimulating contacts were placed at the eighth to twelfth thoracic vertebral (T8–T12) levels for HF-10,^12,17,19–23,27^ HD,^13^ other high frequencies,^20,27^ and Burst SCS modes.^27^ Only two papers described checking for paresthesias in the region of pain by stimulation to aid radiological guidance for positioning the lead for HF-10^12^ or Burst and other high-frequency SCS therapies.^27^ In one study, the authors did not specify the level of SCS lead contacts but stated that “optimal positioning with overlapping paresthesia in painful area” were achieved for Burst SCS (p. 3).^18^

#### Type of trial

Details of the type of SCS trial (percutaneous versus tunneled) were not provided in majority of the studies included in this review. Only two papers mentioned that a trial with HF-10 mode was done percutaneously^12,25^ and another study that used 1.15 kHz for SCS mentioned that the patient had a pre-existing implanted paddle lead.^20^

#### Type and number of leads

The majority of studies included in this review described the type and number of leads used for delivering PF-SCS. Octopolar cylindrical leads (one or two) were used in 55 patients for HF-10^12,21–26^ or Burst and other high frequencies.^18,27^ However, three of the papers in this review did not provide any details about the type or number of leads.^13,17,19^

### Duration of reported follow-up after initiation of SCS

The extent of follow-up had a wide variation in papers included in this review, with a range of less than a day to 24 months. Six of the 13 papers including 41 patients published results of follow-up up to 6 months after initiation of HF-10 SCS,^12,17,19,21^ high-frequency SCS at 1.15 kHz,^20^ and Burst and other high-frequency SCS^27^ and the duration of follow-up was up to 12 months in four publications involving HD^13^ or HF-10.^23,24,26^ In one study on two patients, HF-10 SCS was delivered for only half a day as part of a trial process.^19^ Eight patients in three papers included in this review were followed beyond 12 months with Burst^18^ and HF-10 SCS.^22,25^

### Assessment of pain-related domains and impact of PF-SCS

#### Pain intensity

All of the papers included in this review except one^17^ used the NRS or VAS to measure intensity of pain and the reduction in intensity of pain ranged from 30% to 100%. Of the 62 patients treated with the novel SCS modes, three failed to get analgesic benefit from the 10-kHz HF-SCS. Of note, these three patients had CRPS involving the feet and they also failed an additional week of conventional PB-SCS despite adequate paresthesia coverage of the affected area.^12^ None of the 13 papers reported worsening of intensity or symptoms of CRPS with the novel SCS modes.

#### Type of pain

Characteristics of pain prior to initiation of SCS was assessed in only two studies with the aid of validated tools.^12,27^ Al-Kaisy and colleagues used the painDETECT^12^ as a screening tool to establish whether pain was neuropathic in nature. All six patients with CRPS in this study had high painDETECT scores but the authors did not attempt to correlate these scores with success or failure of HF-SCS. A study by Kriek and colleagues described use of the McGill Pain Questionnaire (MPQ).^27^ The authors described significant reduction in scores on the sensory scale of the MPQ following therapy with all stimulation modes in the trial (40 Hz, 500 Hz, 1200 Hz, Burst) but not with placebo.

#### Features of CRPS and physical function

The impact of PF-SCS on vasomotor and sudomotor symptoms was described in five papers involving use of HF-10^17,21,22,26^ or Burst SCS^18^ with reversal to normal or a halt in progression of these features. None of the studies included in this review mentioned the effect of the novel SCS modes on trophic symptoms and signs of CRPS. The impact of these modes on range of motion ranged from “no worsening” in one report^18^ to an overall improvement^17,21–24,26^ and one case report in which the patient was able to resume pre-injury activities.^25^

#### Quality of life

The impact of the novel SCS modes on quality of life, measured with the EuroQol–five dimensions (EQ-5D) score in two reports that had four patients in total, ranged from an improvement of 101% at 6 months to 341% improvement at 18 months with HF-10 mode.^12,25^ Santarelli and colleagues used the Short Form 36 (SF-36) for this domain with use of HF-10 SCS and they reported significant improvement.^23^ Two other publications commented on improvement in quality of life with high frequency (1.15 kHz) and HF-10 SCS without specifying which tool was used.^20,21^ The severity of pain and the impact on patient’s daily functioning was also investigated with the use of the Brief Pain Inventory (BPI) in one paper with an improvement (reduction) of 45% in the BPI score in all three patients who responded to HF-10 SCS.^12^

#### Analgesic use

Nine of the 13 studies included in this review commented on impact of PF-SCS on analgesic medications. All patients with analgesic benefit from HF-10 SCS were able to lower their daily use of anticonvulsants and opioids, with some patients reporting complete discontinuation of all analgesics with HF-10,^17,21–26^ Burst,^18^ or high-frequency (1.15 kHz) SCS.^20^

#### Psychological function and sleep

Only two studies reported use of validated tools to measure pain-related catastrophization and depression (Pain Catastrophizing Scale [PCS], Center for Epidemiologic Studies–Depression Scale [CES-D])^12,23^ and one study assessed anxiety subjectively.^21^ All three papers involved use of HF-10 SCS and reported significant reduction in PCS and CES-D scores and anxiety. Furthermore, only one study commented on improvement of sleep quality with high-frequency (1.15 kHz) SCS but the authors did not use a validated tool to evaluate this domain.^20^

#### Patients’ preference for type of stimulation

We found only one RCT that compared patients’ preferences about the type of stimulation for CRPS. Four types of stimulation (40 Hz, 500 Hz, 1200 Hz, Burst stimulation) were compared with placebo in this trial. Fifty-two percent of the study patients preferred nonparesthesia SCS (500 Hz, 1200 Hz, Burst stimulation). Though VAS pain scores were lower with preferred stimulation type in responders, all nonparesthesia SCS settings had higher global perceived effect (GPE) scores than placebo.^27^

#### Adverse effects and complications

The adverse effects profile of the novel SCS modes appeared to be similar to conventional PB-SCS and no adverse effects on the central or peripheral nervous system were reported in the publications included in this review. However, Kriek and colleagues identified factors that played a role in patients choosing the type of SCS when they were given an option to trial SCS at 40 Hz, 500 Hz, or 1200 Hz or using Burst stimulation.^27^ Inability to feel paresthesias was identified as a limitation of the novel SCS modes by some patients in this trial. High-frequency SCS (but not Burst) consumes large amounts of energy and it often requires a rechargeable battery with frequent charging and longer duration of charging times compared to conventional PB-SCS. This lack of “recharge burden” with conventional PB-SCS was another reason for some patients preferring this mode in this trial.^27^

### Use of novel SCS modes after failure of PB-SCS

Loss of analgesic effect in patients who previously responded to conventional PB-SCS can have several reasons, including dislocation or malfunction of hardware or development of tolerance. In this review, we came across reports of six patients in four publications who stopped having analgesic benefit with conventional SCS after initial success and despite no lead migration. These patients were reprogrammed with novel SCS modes including HD,^13^ Burst,^18^ high frequency at 1.15 kHz,^20^ and HF-10^26^ settings, with the authors reporting some degree of restoration of analgesic benefit to the patients. We also found one publication in which it was reported that a patient was reprogrammed to HF-10 SCS after a negative conventional SCS-based trial. This patient responded with complete relief of pain, improved range of motion, and resolution of vasomotor and sudomotor changes with a reduction in analgesic requirement after initiation of HF-10 SCS.^21^

## Discussion

This scoping review of the literature on the role of the novel modes of SCS for patients with CRPS is the first of its kind to evaluate evidence for high-frequency, HF-10, Burst, and HD stimulation for this syndrome. Twelve case series or reports and one RCT reported on the use of the novel SCS modes, with failure of conventional PB-SCS being the most common indication for use of these modes. Outcomes were monitored for variable periods of time following initiation of the novel modes.

Though a variety of therapeutic modalities are used to treat CRPS, SCS is one of the few modalities with a high rate of analgesic success for this syndrome.^8^ Conventional SCS is tonic or paresthesia based and its frequency of stimulation ranges between 30 and 80 Hz with patients perceiving nonpainful paresthesias in lieu of otherwise painful sensations. PB-SCS has been shown to deliver good outcomes for some patients with CRPS but many patients have no or unsatisfactory pain relief with this type of SCS or they cannot tolerate the paresthesias. Another therapeutic challenge with conventional SCS is the development of tolerance, leading to diminishing analgesic effect.^8,28^ These limitations have spurred the search for innovations in neuromodulation for treating CRPS. Though not a novel stimulation mode, an excellent example of these efforts is the recently published RCT on stimulation of the dorsal root ganglia (with conventional stimulation of the spinal cord as the comparator) to treat CRPS.^29^ However, this modality relies on a specific anatomic target while using conventional frequencies and subthreshold amplitudes for stimulation.

None of the novel SCS modes are associated with paresthesias, but the postulated mechanisms of action vary for each mode. A majority of papers (11 out of 13) included in our review described the use of high-frequency SCS, an entity that lacks a consensus definition, but any mode with a frequency above 1 kHz is generally accepted in this classification.^11^ Evidence for this SCS mode suggests suppression of mechanical hypersensitivity similar to PB-SCS, while requiring significantly lower intensity of stimulation intensity.^30–32^ The mechanism of action of Burst is also unclear, but proposed explanations include modulation of the medial pain pathway directly by actions on C-fibers synapsing onto lamina I neurons and disruption of synchronous burst firing of the high-threshold fibers that results in inhibition of activation directly related to pain perception.^10^ This is an interesting theory because conventional SCS exerts its effect at the level of the spinal cord and it predominantly modulates the lateral pain pathways.^33^ HD SCS is delivered by increasing the frequency while retaining pulse width of 300–500 ms and keeping the amplitude below the perception threshold. This mode is postulated to transmit higher amounts of electric charge from the SCS electrodes to the neural tissue, thereby increasing analgesic efficacy, without discomfort or damage to the nervous system.^13^

### Technical benefits of using novel SCS modes

Relying on paresthesias to decide on optimal lead positioning in the epidural space and maintaining paresthesias at the location of pain can be challenging and frustrating for patients and health care providers. It requires time and cooperation from a patient who may be quasisedated during the SCS lead placement procedure.^34^ Thus, eliminating reliance on paresthesias can save time and reduce patient discomfort while avoiding the need to adjust stimulation intensity with change in position of the patient.^35^ Use of novel SCS modes also avoids problems due to paresthesias in the painful region or unwanted paresthesias in the nonpainful body locations. These advantages can shorten SCS trial and implantation procedure times. However, this approach requires definitive knowledge of epidural levels of SCS lead placement for pain in specific body regions that provide relief in CRPS. Though the space between the ninth and tenth thoracic vertebra is accepted as the appropriate level for HF-SCS to relieve low back pain, there is a lack of information regarding appropriate levels to relieve pain in the limbs (as is often the presentation in CRPS), especially in the lower limbs.^36^ This may explain the finding that all three patients with CRPS of the feet failed to get any benefit from HF-10 SCS in one of the studies included in our review.^12^ Pending availability of more information regarding “sweet spots” for placing SCS leads to treat CRPS in the limbs, it may be reasonable to check for paresthesias in the region of pain when placing SCS leads with the goal of delivering SCS with one of the novel modes (Burst, high frequency at 500 and 1200 Hz), an approach that was utilized in the RCT by Kriek and colleagues included in this review.^27^ The recently published Effects of Pulse Rate on Clinical Outcomes (PROCO) study has contributed to knowledge regarding optimal lead placement and stimulation frequency for high-frequency modes. This study recruited patients with low back pain (with or without lower limb pain) who were implanted with SCS systems and underwent an 8-week search to identify the best location (sweet spot) of stimulation at 10 kHz within the searched region (T8–T11). Patients who responded to 10-kHz SCS proceeded to double-blind rate randomization to 1-, 4-, 7-, and 10-kHz SCS at the same sweet spot found for 10 kHz in randomized order (4 weeks at each frequency). The authors found that all four frequencies provided equivalent pain and the mean charge per second differed across frequencies, with 1-kHz SCS requiring 60%–70% less charge than higher frequencies.^37^ However, the authors did not specify whether patients with CRPS were enrolled in this study. In another study by Al-Kaisy and colleagues, 24 subjects with predominantly axial low back pain undergoing SCS therapy for failed back surgery syndrome were randomized to sham, 1200 Hz, 3030 Hz, and 5882 Hz with a four-phase crossover design over 12 weeks.^38^ The authors found that SCS at 5882 Hz stimulation produced significant pain relief for axial low back pain compared with lower frequencies and sham stimulation.^38^ Results from these studies suggest the need for more research into optimal “high” frequency for analgesia, especially in patients with CRPS.

### Use of novel SCS modes after failure of conventional SCS

This review shows that patients who are unresponsive to conventional PB-SCS, or those who develop tolerance to it over time, can attain therapeutic benefit by switching to the novel SCS modes.^18,21,24^ However, in the RCT included in this review,^27^ patients were sequentially transitioned from one stimulation mode to another. Though a short washout period (2 days) was provided between application of different SCS modes in this study, a therapeutic bias is possible because the analgesic effects of novel SCS modes could be influenced by previous effects of conventional SCS due to a carryover effect. The same bias could have affected results of two case reports in this review in which HF-10 SCS modes were introduced after failure of conventional SCS, although the follow-up periods exceeded 4 months with preservation of the good results, thereby minimizing the influence of carryover effects.^21,24^ Finally, lack of blinding of patients in these papers to the introduction of novel SCS modes after failure of conventional SCS may have influenced reporting of outcomes by patients.

### Importance of evaluating character of pain using validated tools and the impact of SCS on this domain

Though the Budapest diagnostic criteria for CRPS were accepted by the IASP over 5 years ago, these criteria were used in only two of the papers in this review.^1,12,23^ Further, though CRPS type II is a neuropathic pain syndrome, CRPS type I does not meet the most recent IASP definition of neuropathic pain due to the absence of an identifiable nerve injury.^39^ However, both types of CRPS have a phenotype that is similar to the presentation of neuropathic pain syndromes. Using validated tools to establish that the pain is neuropathic (e.g., Douleur Neuropathique [DN4], Leeds Assessment of Neuropathic Symptoms and Signs [LANSS], painDETECT) and then serial follow-ups with appropriate tools (e.g., Neuropathic Pain Symptom Inventory) to establish whether there is resolution of neuropathic character of the pain with use of novel SCS modes can help build confidence in the ability of these modes to relieve pain in patients with CRPS. Finally, though CRPS has abnormal sensory, sudomotor, vasomotor, trophic, and motor features, the majority of papers in our systematic review only focused on the effect of stimulation on pain and sensory changes. Because all components of CRPS contribute to morbidity, effects of therapies on all domains should be evaluated. Only five papers in this review mentioned the effect of novel SCS modes on nonsensory domains of CRPS.^17,18,21,22,26^

### Importance of evaluating psychological and physical function using validated tools and the impact of SCS on these domains

A majority of the papers included in this review did not describe use of validated tools to evaluate psychological (anxiety, depression, catastrophizing, coping skills) and physical function. This is surprising because the importance of evaluating pain-related domains in clinical and research settings is established and validated tools to evaluate these domains are also widely available.^40^ SCS is a resource-intensive intervention and comprehensive evaluation of outcomes to establish its impact in CRPS is essential. Recently published studies have also cast doubts on the assumption that benefit from high-frequency SCS is enhanced proportional to the frequency of stimulation.^37^ Thus, multidomain monitoring of features of CRPS and estimation of cost–benefit ratios are essential for establishing efficacy and effectiveness of novel SCS modes.

### Adverse effects and safety of novel SCS modes

None of the papers included in this review reported any serious adverse effects or safety issues when novel SCS modes were used in patients with CRPS. Animal and clinical studies have been published on the possible adverse effects of HF-10 on the spinal cord and these studies report a safety profile similar to that of conventional SCS.^11^ However, HF-10 and HD potentially deliver electrical charge of a higher magnitude or with a higher efficacy to the spinal cord^10^ and require frequent recharging of the implanted pulse generator. These modes are also fairly new to clinical practice and their long-term adverse effects, if any, are unknown.

We have provided recommendations for data elements to be included in studies on novel SCS modes for patients with CRPS in Table 3.

**Table 3.** Recommendations for data elements to be included in studies on novel SCS modes for patients with CRPS.

1. Patient-related and CRPS-related data 2. Age 3. Sex 4. BMI 5. CRPS diagnosis confirmed as per IASP Budapest criteria including details of the four domains: sensory, motor, vasomotor, sudomotor 6. Type of trauma that preceeded CRPS 7. Duration of CRPS 8. Analgesic usage 9. Ability to work and or participate in activities important for the patient
**Data related to SCS implant and mode**
● Stimulation frequency, amplitude or voltage, pulse width, pulse shape ● Details of the implanted lead in the epidural space and electrode(s) stimulated in relation to vertebral levels
**Domains to be assessed at baseline and at serial post-SCS implantation follow-up visits**
● Pain-related: NRS, BPI, neuropathic pain score (e.g., DN4) ● Pain-related domains: ● Functional status (e.g., SF-36, PSQ-3) ● Psychological status (e.g., PCS, GAD-7, PHQ-9) ● Physical status (e.g., LEFS) ○ Analgesic usage ○ Effect of SCS on CRPS-associated domains ○ SCS stimulation mode preference ○ Adverse effects or events related to SCS ○ Ability to work and or participate in activities important for the patient

SCS = spinal cord stimulation; CRPS = complex regional pain syndrome; BMI = body mass index; IASP = International Association for the Study of Pain; NRS = numerical rating scale; BPI = Brief Pain Inventory; DN4 = Douleur Neuropathique (four questions); SF-36 = Short Form survey (36 items); PSQ-3 = Pain and Sleep Questionnaire (three questions); PCS = Pain Catastrophizing Scale; GAD-7 = Generalized Anxiety Questionnaire (seven questions); PHQ-9 = Patient Health Questionnaire for Depression (nine questions); LEFS = Lower Extremity Function Scale.

There are some limitations of our review. Although we conducted a comprehensive search of literature, only 13 papers on novel SCS modes for CRPS with a total of 62 patients were identified. The quality of evidence supporting use of these modes was modest and the papers included in this review were seven case reports or series^12,13,17–21^ and five conference abstracts.^22–26^ Case series or reports occupies a low rung in the hierarchy for evidence-based medicine because observational data are prone to various types of biases, including expectation, measurement, and reporting biases.^41^ Further, abstracts are often not considered to be peer reviewed. A case in point is the absence of papers in the literature that report failure of novel SCS modes in CRPS. The wide range of follow-up periods in papers included in this review also makes it difficult to gauge the longevity of benefit from these modes. One of the case series included in this review had an observation period of only half a day following initiation of the novel SCS modes but the authors acknowledged that their study was not designed to test the long-term effect of such therapy. We agree with the authors reasoning about the observation that patients experienced benefit with high-frequency stimulation suggests a strong effect of high-frequency SCS therapy. Only 30% of patients included in this review were followed beyond 6 months following initiation of novel SCS modes and a few papers reported outcomes at 12 months or beyond following initiation of these modes.^10,18,22–25^ Follow-up for a year and beyond is usually required to assess longevity of analgesic effects, as well as the effect of novel SCS modes on other CRPS symptoms and the possible long-term adverse effects and failure resulting in explantation of the system.

## Conclusions

In our scoping review, we found papers that reported benefit from HF-10, Burst, and HD SCS modes in patients with CRPS. However, the low quality of this evidence precludes any definitive conclusions as to whether these novel SCS modes provide better therapeutic efficacy over the long term compared to conventional paresthesia-based modes in patients with CRPS. Rigorous head-to-head comparisons of the novel SCS modes against each other and against conventional paresthesia-based modes in patients with a diagnosis of CRPS established as per IASP criteria are required. Evaluation of all pain-related domains over a period of time (at least one year) in these trials is essential to build a high-quality pool of evidence for the role of novel SCS modes in SCS.

## Supplementary Material

Supplemental Material
