# Supplementary material for: A scoping review of novel spinal cord stimulation modes for complex regional pain syndrome
Source: Can J Pain. 2019 Mar 5;3(1):33–48. doi: 10.1080/24740527.2019.1574536 (PMC8730659; doi:10.1080/24740527.2019.1574536)
Supplement: Supplemental Material [file UCJP_A_1574536_SM9227.zip › Appendix 2 CJP literature search results.docx]

**
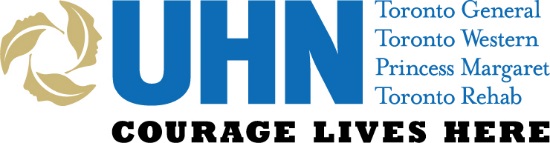
**

**Appendix 2.**

# UHN Health Sciences Libraries

# Literature Search Results

For: Drs Y Hoydonckx & A Bhatia

Department: Anesthesia/Pain (TWH)

Date Completed: Thursday, Sept. 21, 2017

Tel:

Fax:

Email:

**Attached is your search for**:

1. Systematic Review FINAL: High frequency spinal cord stimulation + complex regional pain syndrome; limited to humans (where possible).

**The databases searched were**:

1. [Medline](#MEDLINE); 2. [Medline In-Process / ePubs](#Medline_In_Process_Epub_Ahead_of_Print); 3. [Embase](#Embase); 4. [CCTR](#Cochrane_Central_Register_of_Controlled); 5. [CDSR](#Cochrane_Database_of_Systematic_Reviews); 6. [PubMed-NOT-Medline;](#PubMed_NOT_Medline) 7. [Biosys Previews](#Biosys_Previews); 8. [Scopus](#Scopus); 9. [ClinicalTrials.Gov](#ClinicalTrials_Gov); 10. [Google Scholar](#Google_Scholar) (search string only); 11. [AHRQ HRA / EPC](#AHRQ); 12. [NIHR](#NIHR_Health_Technology_Assessment_Progra).

**RESULTS & STRATEGY USED**: *see following*

**Search Completed By:** Marina Englesakis, Information Specialist

You may contact me either by telephone at (416) 340-4800 x3022 or via e-mail at [*marina.englesakis@uhn.ca*](mailto:marina.englesakis@uhn.ca)*.*

It is important that you are satisfied with your search results.

If you have any questions regarding this search, or if the results were not satisfactory, please do not hesitate in contacting me.

To request items not available in our library system, an INTERLIBRARY LOAN REQUEST FORM can be obtained from the library’s circulation desk or through the Virtual Library (<http://www.uhn.ca/Education/libraries/ill.asp> ). Any questions regarding our Document Delivery Service can be directed to Walter by telephone at 416-340-4121 or by email at [Walter.Schmanda@uhn.ca](mailto:Walter.Schmanda@uhn.ca).

For any other circulation inquiries:

Toronto General Hospital Library: (416) 340-3429

Toronto Western Hospital Library: (416) 603-5750

Toronto Rehab Library: (416) 597-3422, ext. 3050

Princess Margaret Library: (416) 946-4482

2017-09-21 AB YH -FINAL- High-frequency spinal cord stimulation in Complex Regional Pain Syndrome - Medl

High-frequency spinal cord stimulation AND Complex Regional Pain Syndrome (many synonyms added) NB: “Studies or Costs” block removed as per client email, Sept 18, 2017. Thursday September 21 2017

Ovid MEDLINE (R) 1946 to September Week 2 2017

| # | Searches | Results |
| --- | --- | --- |
| 1 | Spinal Cord Stimulation/ [ New MeSH as of 2013] | 624 |
| 2 | Electric Stimulation Therapy/ | 19497 |
| 3 | Electrodes, Implanted/ | 19337 |
| 4 | "10 kHz".mp,kw. | 1307 |
| 5 | "10 kHz".mp,kw. | 1307 |
| 6 | "1000 Hertz".mp,kw. | 7 |
| 7 | "1000 Hz".mp,kw. | 1667 |
| 8 | "10-kHz high-frequenc*".mp,kw. | 7 |
| 9 | "500 Hertz".mp,kw. | 3 |
| 10 | "500 Hz".mp,kw. | 2328 |
| 11 | (HF?? adj4 stimulat*).mp,kw. | 1436 |
| 12 | (HF10 adj4 stimulat*).mp,kw. | 9 |
| 13 | (high frequenc* adj3 stimulat*).mp,kw. | 4376 |
| 14 | (high-frequenc* adj2 peripheral adj2 nerve? adj3 stimulat*).mp,kw. | 7 |
| 15 | (high-frequenc* adj4 block*).mp,kw. | 216 |
| 16 | (high-frequenc* adj4 waveform*).mp. | 76 |
| 17 | (high-frequency adj3 par?esthesia-free).mp,kw. | 4 |
| 18 | (kilohertz adj5 stimulat*).mp,kw. | 18 |
| 19 | (kilohertz frequenc* adj2 epidural* adj2 stimulat*).mp,kw. | 1 |
| 20 | (maxim?? adj2 voltage? adj2 stimulat*).mp,kw. | 11 |
| 21 | (stimulat* electrode? adj4 peripheral nerve?).mp,kw. | 11 |
| 22 | burst.mp,kw. | 40185 |
| 23 | HF10 SCS.mp,kw. | 7 |
| 24 | HF10 Therapy*.mp,kw. | 5 |
| 25 | HFSCS.mp,kw. | 62 |
| 26 | high-frequency SCS.mp,kw. | 10 |
| 27 | High-frequency therap*.mp,kw. | 29 |
| 28 | kilohertz alternating current?.mp,kw. | 1 |
| 29 | kilohertz electrical nerve block*.mp,kw. | 1 |
| 30 | neuromodulation?.mp,kw. | 4259 |
| 31 | neuro-modulation?.mp,kw. | 38 |
| 32 | paraesthesia-free.mp,kw. | 1 |
| 33 | paresthesia-free.mp,kw. | 17 |
| 34 | or/1-33 [ High Frequency Spinal Cord Stimulation ] | 88885 |
| 35 | exp Complex Regional Pain Syndromes/ [MeSH] | 5348 |
| 36 | Causalgia/ [MeSH] | 687 |
| 37 | Reflex Sympathetic Dystrophy/ [MeSH] | 3629 |
| 38 | CRPS.mp. | 1627 |
| 39 | "CRPS 1".mp. | 90 |
| 40 | "CRPS 2".mp. | 4 |
| 41 | "CRPS type 1".mp. | 69 |
| 42 | "CRPS type 2".mp. | 10 |
| 43 | algodystroph*.mp. | 527 |
| 44 | Causalgia??.mp. | 892 |
| 45 | Complex Regional Pain Syndrome?.mp. | 2355 |
| 46 | Morbus Sudeck.mp. | 34 |
| 47 | neuralgic shoulder? amyotroph*.mp. | 14 |
| 48 | (posttraumatic adj1 dystroph*).mp. | 26 |
| 49 | (post-traumatic adj1 dystroph*).mp. | 19 |
| 50 | (posttraumatic adj1 osteoporos#s).mp. | 12 |
| 51 | (post-traumatic adj1 osteoporos#s).mp. | 14 |
| 52 | Reflex Sympathetic Dystroph*.mp. | 4065 |
| 53 | shoulder arm syndrome?.mp. | 52 |
| 54 | shoulder hand syndrome?.mp. | 320 |
| 55 | (sudeck?? adj1 atroph*).mp. | 170 |
| 56 | (sympathetic adj2 dystroph*).mp. | 4136 |
| 57 | sympathetic dystroph* syndrome?.mp. | 237 |
| 58 | sympathetic reflex dystroph*.mp. | 66 |
| 59 | Sudeck PH.pn. | 2 |
| 60 | or/35-59 [ Complex Regional Pain Syndromes & related terms ] | 6983 |
| 61 | 34 and 60 [ HF SCS and Complex Regional Pain Syndrome ] | 328 |
| 62 | exp animals/ not (exp animals/ and humans/) | 4584143 |
| 63 | 61 not 62 | 325 |
| 64 | limit 61 to human | 323 |
| 65 | 63 or 64 | 325 |
| 66 | remove duplicates from 65 | 310 |

Ovid MEDLINE(R) Epub Ahead of Print and In-Process & Other Non-Indexed Citations September 20, 2017

| # | Searches | Results |
| --- | --- | --- |
| 1 | Spinal Cord Stimulation/ [ New MeSH as of 2013] | 0 |
| 2 | Electric Stimulation Therapy/ | 3 |
| 3 | Electrodes, Implanted/ | 0 |
| 4 | "10 kHz".mp,kw. | 535 |
| 5 | "10 kHz".mp,kw. | 535 |
| 6 | "1000 Hertz".mp,kw. | 4 |
| 7 | "1000 Hz".mp,kw. | 168 |
| 8 | "10-kHz high-frequenc*".mp,kw. | 3 |
| 9 | "500 Hertz".mp,kw. | 2 |
| 10 | "500 Hz".mp,kw. | 295 |
| 11 | (HF?? adj4 stimulat*).mp,kw. | 144 |
| 12 | (HF10 adj4 stimulat*).mp,kw. | 1 |
| 13 | (high frequenc* adj3 stimulat*).mp,kw. | 331 |
| 14 | (high-frequenc* adj2 peripheral adj2 nerve? adj3 stimulat*).mp,kw. | 0 |
| 15 | (high-frequenc* adj4 block*).mp,kw. | 17 |
| 16 | (high-frequenc* adj4 waveform*).mp. | 10 |
| 17 | (high-frequency adj3 par?esthesia-free).mp,kw. | 0 |
| 18 | (kilohertz adj5 stimulat*).mp,kw. | 6 |
| 19 | (kilohertz frequenc* adj2 epidural* adj2 stimulat*).mp,kw. | 0 |
| 20 | (maxim?? adj2 voltage? adj2 stimulat*).mp,kw. | 1 |
| 21 | (stimulat* electrode? adj4 peripheral nerve?).mp,kw. | 3 |
| 22 | burst.mp,kw. | 4010 |
| 23 | HF10 SCS.mp,kw. | 0 |
| 24 | HF10 Therapy*.mp,kw. | 2 |
| 25 | HFSCS.mp,kw. | 24 |
| 26 | high-frequency SCS.mp,kw. | 6 |
| 27 | High-frequency therap*.mp,kw. | 4 |
| 28 | kilohertz alternating current?.mp,kw. | 0 |
| 29 | kilohertz electrical nerve block*.mp,kw. | 0 |
| 30 | neuromodulation?.mp,kw. | 1256 |
| 31 | neuro-modulation?.mp,kw. | 14 |
| 32 | paraesthesia-free.mp,kw. | 0 |
| 33 | paresthesia-free.mp,kw. | 6 |
| 34 | or/1-33 [ High Frequency Spinal Cord Stimulation ] | 6630 |
| 35 | exp Complex Regional Pain Syndromes/ [MeSH] | 0 |
| 36 | Causalgia/ [MeSH] | 0 |
| 37 | Reflex Sympathetic Dystrophy/ [MeSH] | 0 |
| 38 | CRPS.mp. | 344 |
| 39 | "CRPS 1".mp. | 19 |
| 40 | "CRPS 2".mp. | 3 |
| 41 | "CRPS type 1".mp. | 20 |
| 42 | "CRPS type 2".mp. | 3 |
| 43 | algodystroph*.mp. | 23 |
| 44 | Causalgia??.mp. | 45 |
| 45 | Complex Regional Pain Syndrome?.mp. | 450 |
| 46 | Morbus Sudeck.mp. | 5 |
| 47 | neuralgic shoulder? amyotroph*.mp. | 0 |
| 48 | (posttraumatic adj1 dystroph*).mp. | 0 |
| 49 | (post-traumatic adj1 dystroph*).mp. | 4 |
| 50 | (posttraumatic adj1 osteoporos#s).mp. | 0 |
| 51 | (post-traumatic adj1 osteoporos#s).mp. | 0 |
| 52 | Reflex Sympathetic Dystroph*.mp. | 93 |
| 53 | shoulder arm syndrome?.mp. | 2 |
| 54 | shoulder hand syndrome?.mp. | 5 |
| 55 | (sudeck?? adj1 atroph*).mp. | 7 |
| 56 | (sympathetic adj2 dystroph*).mp. | 101 |
| 57 | sympathetic dystroph* syndrome?.mp. | 10 |
| 58 | sympathetic reflex dystroph*.mp. | 3 |
| 59 | Sudeck PH.pn. | 0 |
| 60 | or/35-59 [ Complex Regional Pain Syndromes & related terms ] | 640 |
| 61 | 34 and 60 [ HF SCS and Complex Regional Pain Syndrome ] | 18 |

Embase Classic+Embase 1947 to 2017 September 20

| # | Searches | Results |
| --- | --- | --- |
| 1 | exp Complex Regional Pain Syndromes/ | 8963 |
| 2 | Causalgia/ | 1181 |
| 3 | Reflex Sympathetic Dystrophy/ | 2285 |
| 4 | exp complex regional pain syndrome/ [ Embase ] | 8963 |
| 5 | exp complex regional pain syndrome type i/ [ Embase ] | 5534 |
| 6 | algodystrophy/ [ Embase ] | 809 |
| 7 | posttraumatic osteoporosis/ [ Embase ] | 785 |
| 8 | sympathetic dystrophy/ [ Embase ] | 1378 |
| 9 | complex regional pain syndrome type II/ [ Embase ] | 302 |
| 10 | Complex Regional Pain Syndrome?.mp. | 6924 |
| 11 | CRPS.mp. | 3071 |
| 12 | "CRPS 1".mp. | 174 |
| 13 | "CRPS 2".mp. | 24 |
| 14 | "CRPS type 1".mp. | 147 |
| 15 | "CRPS type 2".mp. | 30 |
| 16 | (posttraumatic adj1 dystroph*).mp. | 43 |
| 17 | (post-traumatic adj1 dystroph*).mp. | 39 |
| 18 | (posttraumatic adj1 osteoporo*).mp. | 796 |
| 19 | (post-traumatic adj1 osteoporo*).mp. | 52 |
| 20 | (Sudeck?? adj1 atroph*).mp. | 292 |
| 21 | (sympathetic adj2 dystroph*).mp. | 2996 |
| 22 | algodystroph*.mp. | 1220 |
| 23 | Causalgia??.mp. | 1509 |
| 24 | Morbus Sudeck.mp. | 41 |
| 25 | neuralgic shoulder? amyotroph*.mp. | 14 |
| 26 | Reflex Sympathetic Dystroph*.mp. | 2381 |
| 27 | shoulder arm syndrome?.mp. | 125 |
| 28 | shoulder hand syndrome?.mp. | 813 |
| 29 | sympathetic dystroph* syndrome?.mp. | 294 |
| 30 | sympathetic reflex dystroph*.mp. | 107 |
| 31 | or/1-30 [ Complex Regional Pain Syndromes & related terms ] | 12493 |
| 32 | exp high frequency electrotherapy/ | 6884 |
| 33 | electrode implant/ | 2128 |
| 34 | electrode implantation/ | 907 |
| 35 | electrostimulation/ | 82768 |
| 36 | electrotherapy/ | 527 |
| 37 | nerve stimulation/ | 32825 |
| 38 | neuromodulation/ | 34432 |
| 39 | spinal cord stimulation/ | 5393 |
| 40 | spinal cord stimulator/ | 440 |
| 41 | spinal ganglion/ and (stimulat* or neuromod* or neurostim*).mp,kw. | 4391 |
| 42 | ">10 kHz".mp,kw. | 1735 |
| 43 | ">1000 Hertz".mp,kw. | 8 |
| 44 | ">1000 Hz".mp,kw. | 2534 |
| 45 | ">10kHz".mp,kw. | 116 |
| 46 | "10 kHz".mp,kw. | 1735 |
| 47 | "1000 Hertz".mp,kw. | 8 |
| 48 | "1000 Hz".mp,kw. | 2534 |
| 49 | "10-kHz high-frequenc*".mp,kw. | 18 |
| 50 | "10kHz".mp,kw. | 116 |
| 51 | "500 Hertz".mp,kw. | 12 |
| 52 | "500 Hz".mp,kw. | 3214 |
| 53 | ((spin??? adj1 gangli???) and (stimulat* or neuromod* or neurostim*)).mp,kw. | 4453 |
| 54 | (burst and (stimulat* or neuromod* or neurostim*)).mp,kw. | 15634 |
| 55 | ((dorsal adj1 root? adj1 gangli*) and stim*).mp,kw. | 4209 |
| 56 | (HF?? adj4 stimulat*).mp,kw. | 2244 |
| 57 | (HF10 adj4 stimulat*).mp,kw. | 38 |
| 58 | (high frequenc* adj3 stimulat*).mp,kw. | 6179 |
| 59 | (high-frequenc* adj2 peripheral adj2 nerve? adj3 stimulat*).mp,kw. | 7 |
| 60 | (high-frequenc* adj4 block*).mp,kw. | 287 |
| 61 | (high-frequenc* adj4 waveform*).mp. | 128 |
| 62 | (high-frequency adj3 par?esthesia-free).mp,kw. | 10 |
| 63 | (kilohertz adj5 stimulat*).mp,kw. | 35 |
| 64 | (kilohertz frequenc* adj2 epidural* adj2 stimulat*).mp,kw. | 1 |
| 65 | (maxim?? adj2 voltage? adj2 stimulat*).mp,kw. | 19 |
| 66 | (novel and (stimulat* or neuromod* or neurostim*)).mp,kw. | 112827 |
| 67 | (spinal adj2 cord? adj2 stimulat*).mp,kw. | 7243 |
| 68 | (stimulat* electrode? adj4 peripheral nerve?).mp,kw. | 16 |
| 69 | HF10SCS.mp,kw. | 0 |
| 70 | HF10 SCS.mp,kw. | 27 |
| 71 | HF10 Therap*.mp,kw. | 48 |
| 72 | HFSCS.mp,kw. | 121 |
| 73 | HF-SCS.mp,kw. | 71 |
| 74 | high-frequency SCS.mp,kw. | 54 |
| 75 | High-frequency therap*.mp,kw. | 53 |
| 76 | kilohertz alternating current?.mp,kw. | 1 |
| 77 | kilohertz electrical nerve block*.mp,kw. | 1 |
| 78 | kilohertz electrical neural block*.mp,kw. | 0 |
| 79 | neuromodulation?.mp,jw,kw. | 39922 |
| 80 | neuro-modulation?.mp,kw. | 103 |
| 81 | paraesthesia-free.mp,kw. | 14 |
| 82 | paresthesia-free.mp,kw. | 64 |
| 83 | Spinal Cord Stimulation/ [ New MeSH as of 2013] | 5393 |
| 84 | Electric Stimulation Therapy/ [MeSH] | 469 |
| 85 | Electrodes, Implanted/ [MeSH] | 902 |
| 86 | or/32-85 [ High-Frequency Stimulation ] | 295388 |
| 87 | 31 and 86 [ CRPS + High Frequency Stimulation ] | 1142 |
| 88 | (exp animals/ or exp animal experimentation/ or nonhuman/) not ((exp animals/ or exp animal experimentation/ or nonhuman/) and exp human/) | 6642507 |
| 89 | 87 not 88 | 1111 |
| 90 | limit 87 to human | 1013 |
| 91 | 89 or 90 | 1111 |
| 92 | remove duplicates from 91 | 1086 |

EBM Reviews - Cochrane Central Register of Controlled Trials August 2017

| # | Searches | Results |
| --- | --- | --- |
| 1 | exp Complex Regional Pain Syndromes/ | 195 |
| 2 | Causalgia/ | 11 |
| 3 | Reflex Sympathetic Dystrophy/ | 135 |
| 4 | exp complex regional pain syndrome/ [ Embase ] | 0 |
| 5 | exp complex regional pain syndrome type i/ [ Embase ] | 135 |
| 6 | algodystrophy/ [ Embase ] | 135 |
| 7 | posttraumatic osteoporosis/ [ Embase ] | 0 |
| 8 | sympathetic dystrophy/ [ Embase ] | 0 |
| 9 | complex regional pain syndrome type II/ [ Embase ] | 11 |
| 10 | Complex Regional Pain Syndrome?.mp. | 281 |
| 11 | CRPS.mp. | 169 |
| 12 | "CRPS 1".mp. | 21 |
| 13 | "CRPS 2".mp. | 0 |
| 14 | "CRPS type 1".mp. | 15 |
| 15 | "CRPS type 2".mp. | 0 |
| 16 | (posttraumatic adj1 dystroph*).mp. | 2 |
| 17 | (post-traumatic adj1 dystroph*).mp. | 3 |
| 18 | (posttraumatic adj1 osteoporo*).mp. | 7 |
| 19 | (post-traumatic adj1 osteoporo*).mp. | 0 |
| 20 | (Sudeck?? adj1 atroph*).mp. | 2 |
| 21 | (sympathetic adj2 dystroph*).mp. | 185 |
| 22 | algodystroph*.mp. | 28 |
| 23 | Causalgia??.mp. | 20 |
| 24 | Morbus Sudeck.mp. | 0 |
| 25 | neuralgic shoulder? amyotroph*.mp. | 0 |
| 26 | Reflex Sympathetic Dystroph*.mp. | 179 |
| 27 | shoulder arm syndrome?.mp. | 19 |
| 28 | shoulder hand syndrome?.mp. | 40 |
| 29 | sympathetic dystroph* syndrome?.mp. | 10 |
| 30 | sympathetic reflex dystroph*.mp. | 2 |
| 31 | or/1-30 [ Complex Regional Pain Syndromes & related terms ] | 478 |
| 32 | exp high frequency electrotherapy/ | 0 |
| 33 | electrode implant/ | 0 |
| 34 | electrode implantation/ | 0 |
| 35 | electrostimulation/ | 0 |
| 36 | electrotherapy/ | 1557 |
| 37 | nerve stimulation/ | 0 |
| 38 | neuromodulation/ | 0 |
| 39 | spinal cord stimulation/ | 27 |
| 40 | spinal cord stimulator/ | 0 |
| 41 | spinal ganglion/ and (stimulat* or neuromod* or neurostim*).mp,kw. | 4 |
| 42 | ">10 kHz".mp,kw. | 40 |
| 43 | ">1000 Hertz".mp,kw. | 1 |
| 44 | ">1000 Hz".mp,kw. | 99 |
| 45 | ">10kHz".mp,kw. | 5 |
| 46 | "10 kHz".mp,kw. | 40 |
| 47 | "1000 Hertz".mp,kw. | 1 |
| 48 | "1000 Hz".mp,kw. | 99 |
| 49 | "10-kHz high-frequenc*".mp,kw. | 7 |
| 50 | "10kHz".mp,kw. | 5 |
| 51 | "500 Hertz".mp,kw. | 1 |
| 52 | "500 Hz".mp,kw. | 89 |
| 53 | ((spin??? adj1 gangli???) and (stimulat* or neuromod* or neurostim*)).mp,kw. | 19 |
| 54 | (burst and (stimulat* or neuromod* or neurostim*)).mp,kw. | 563 |
| 55 | ((dorsal adj1 root? adj1 gangli*) and stim*).mp,kw. | 27 |
| 56 | (HF?? adj4 stimulat*).mp,kw. | 170 |
| 57 | (HF10 adj4 stimulat*).mp,kw. | 3 |
| 58 | (high frequenc* adj3 stimulat*).mp,kw. | 216 |
| 59 | (high-frequenc* adj2 peripheral adj2 nerve? adj3 stimulat*).mp,kw. | 2 |
| 60 | (high-frequenc* adj4 block*).mp,kw. | 11 |
| 61 | (high-frequenc* adj4 waveform*).mp. | 4 |
| 62 | (high-frequency adj3 par?esthesia-free).mp,kw. | 1 |
| 63 | (kilohertz adj5 stimulat*).mp,kw. | 2 |
| 64 | (kilohertz frequenc* adj2 epidural* adj2 stimulat*).mp,kw. | 1 |
| 65 | (maxim?? adj2 voltage? adj2 stimulat*).mp,kw. | 4 |
| 66 | (novel and (stimulat* or neuromod* or neurostim*)).mp,kw. | 2084 |
| 67 | (spinal adj2 cord? adj2 stimulat*).mp,kw. | 353 |
| 68 | (stimulat* electrode? adj4 peripheral nerve?).mp,kw. | 1 |
| 69 | HF10SCS.mp,kw. | 0 |
| 70 | HF10 SCS.mp,kw. | 2 |
| 71 | HF10 Therap*.mp,kw. | 14 |
| 72 | HFSCS.mp,kw. | 2 |
| 73 | HF-SCS.mp,kw. | 1 |
| 74 | high-frequency SCS.mp,kw. | 5 |
| 75 | High-frequency therap*.mp,kw. | 9 |
| 76 | kilohertz alternating current?.mp,kw. | 0 |
| 77 | kilohertz electrical nerve block*.mp,kw. | 0 |
| 78 | kilohertz electrical neural block*.mp,kw. | 0 |
| 79 | neuromodulation?.mp,jw,kw. | 823 |
| 80 | neuro-modulation?.mp,kw. | 4 |
| 81 | paraesthesia-free.mp,kw. | 2 |
| 82 | paresthesia-free.mp,kw. | 15 |
| 83 | Spinal Cord Stimulation/ [ New MeSH as of 2013] | 27 |
| 84 | Electric Stimulation Therapy/ [MeSH] | 1557 |
| 85 | Electrodes, Implanted/ [MeSH] | 363 |
| 86 | or/32-85 [ High-Frequency Stimulation ] | 5732 |
| 87 | 31 and 86 [ CRPS + High Frequency Stimulation ] | 44 |
| 88 | remove duplicates from 87 | 43 |
| 89 | limit 88 to medline records | 20 |
| 90 | limit 88 to embase records | 22 |
| 91 | 89 or 90 | 42 |
| 92 | 88 not 91 | 1 |

EBM Reviews - Cochrane Database of Systematic Reviews 2005 to September 20, 2017

| # | Searches | Results |
| --- | --- | --- |
| 1 | Complex Regional Pain Syndrome?.mp. | 73 |
| 2 | CRPS.mp. | 40 |
| 3 | "CRPS 1".mp. | 4 |
| 4 | "CRPS 2".mp. | 0 |
| 5 | "CRPS type 1".mp. | 4 |
| 6 | "CRPS type 2".mp. | 0 |
| 7 | (posttraumatic adj1 dystroph*).mp. | 0 |
| 8 | (post-traumatic adj1 dystroph*).mp. | 2 |
| 9 | (posttraumatic adj1 osteoporo*).mp. | 1 |
| 10 | (post-traumatic adj1 osteoporo*).mp. | 1 |
| 11 | (Sudeck?? adj1 atroph*).mp. | 10 |
| 12 | (sympathetic adj2 dystroph*).mp. | 31 |
| 13 | algodystroph*.mp. | 11 |
| 14 | Causalgia??.mp. | 9 |
| 15 | Morbus Sudeck.mp. | 0 |
| 16 | neuralgic shoulder? amyotroph*.mp. | 0 |
| 17 | Reflex Sympathetic Dystroph*.mp. | 29 |
| 18 | shoulder arm syndrome?.mp. | 0 |
| 19 | shoulder hand syndrome?.mp. | 11 |
| 20 | sympathetic dystroph* syndrome?.mp. | 3 |
| 21 | sympathetic reflex dystroph*.mp. | 2 |
| 22 | or/1-21 [ Complex Regional Pain Syndromes & related terms ] | 95 |
| 23 | ">10 kHz".mp,kw. | 3 |
| 24 | ">1000 Hertz".mp,kw. | 0 |
| 25 | ">1000 Hz".mp,kw. | 2 |
| 26 | ">10kHz".mp,kw. | 0 |
| 27 | "10 kHz".mp,kw. | 3 |
| 28 | "1000 Hertz".mp,kw. | 0 |
| 29 | "1000 Hz".mp,kw. | 2 |
| 30 | "10-kHz high-frequenc*".mp,kw. | 0 |
| 31 | "10kHz".mp,kw. | 0 |
| 32 | "500 Hertz".mp,kw. | 0 |
| 33 | "500 Hz".mp,kw. | 3 |
| 34 | ((spin??? adj1 gangli???) and (stimulat* or neuromod* or neurostim*)).mp,kw. | 3 |
| 35 | (burst and (stimulat* or neuromod* or neurostim*)).mp,kw. | 35 |
| 36 | ((dorsal adj1 root? adj1 gangli*) and stim*).mp,kw. | 16 |
| 37 | (HF?? adj4 stimulat*).mp,kw. | 6 |
| 38 | (HF10 adj4 stimulat*).mp,kw. | 0 |
| 39 | (high frequenc* adj3 stimulat*).mp,kw. | 19 |
| 40 | (high-frequenc* adj2 peripheral adj2 nerve? adj3 stimulat*).mp,kw. | 0 |
| 41 | (high-frequenc* adj4 block*).mp,kw. | 2 |
| 42 | (high-frequenc* adj4 waveform*).mp. | 1 |
| 43 | (high-frequency adj3 par?esthesia-free).mp,kw. | 0 |
| 44 | (kilohertz adj5 stimulat*).mp,kw. | 0 |
| 45 | (kilohertz frequenc* adj2 epidural* adj2 stimulat*).mp,kw. | 0 |
| 46 | (maxim?? adj2 voltage? adj2 stimulat*).mp,kw. | 1 |
| 47 | (novel and (stimulat* or neuromod* or neurostim*)).mp,kw. | 196 |
| 48 | (spinal adj2 cord? adj2 stimulat*).mp,kw. | 17 |
| 49 | (stimulat* electrode? adj4 peripheral nerve?).mp,kw. | 0 |
| 50 | HF10SCS.mp,kw. | 0 |
| 51 | HF10 SCS.mp,kw. | 0 |
| 52 | HF10 Therap*.mp,kw. | 0 |
| 53 | HFSCS.mp,kw. | 0 |
| 54 | HF-SCS.mp,kw. | 0 |
| 55 | high-frequency SCS.mp,kw. | 0 |
| 56 | High-frequency therap*.mp,kw. | 1 |
| 57 | kilohertz alternating current?.mp,kw. | 0 |
| 58 | kilohertz electrical nerve block*.mp,kw. | 0 |
| 59 | kilohertz electrical neural block*.mp,kw. | 0 |
| 60 | neuromodulation?.mp,jw,kw. | 35 |
| 61 | neuro-modulation?.mp,kw. | 4 |
| 62 | paraesthesia-free.mp,kw. | 0 |
| 63 | paresthesia-free.mp,kw. | 0 |
| 64 | or/23-63 [ High-Frequency Stimulation ] | 308 |
| 65 | 22 and 64 [ CRPS + High Frequency Stimulation ] | 17 |
| 66 | limit 65 to full systematic reviews | 16 |

PubMed-NOT-Medline

| Search | Query | Items found |
| --- | --- | --- |
| #5 | Search ((((((Complex Regional Pain Syndromes[MeSH]) OR (Causalgia[MeSH]) OR (Reflex Sympathetic Dystrophy[MeSH]) OR (CRPS[tw]) OR ("CRPS 1"[tw]) OR ("CRPS 2"[tw]) OR ("CRPS type 1"[tw]) OR ("CRPS type 2"[tw]) OR (algodystroph*[tw]) OR (causalgia[tw]) OR (causalgias[tw]) OR (“Complex Regional Pain Syndrome”[tw]) OR (“Complex Regional Pain Syndromes”“[tw]) OR (Morbus Sudeck[tw]) OR (neuralgic shoulder amyotroph*[tw]) OR (posttraumatic dystrophy[tw]) OR (posttraumatic dystrophies[tw]) OR (post-traumatic dystrophy[tw]) OR (post-traumatic dystrophies[tw]) OR (posttraumatic osteoporoses[tw]) OR (posttraumatic osteoporosis[tw]) OR (post-traumatic osteoporoses[tw]) OR (post-traumatic osteoporosis[tw]) OR (Reflex Sympathetic[tw]) OR (Reflex Sympathetic Dystrophy[tw]) OR (Reflex Sympathetic Dystrophies[tw]) OR (shoulder arm syndrome[tw]) OR (shoulder arm syndromes[tw]) OR (shoulder hand syndrome[tw]) OR (shoulder hand syndromes[tw]) OR (sudeck* atrophy[tw]) OR (sudeck* atrophy*[tw]) OR (sympathetic dystrophy[tw]) OR (sympathetic dystrophies[tw]) OR (sympathetic dystroph* syndrome[tw]) OR (sympathetic dystroph* syndromes[tw]) OR (sympathetic reflex dystroph*[tw]) OR (Sudeck PH[all fields]))))) AND ((((Spinal Cord Stimulation[MeSH]) OR (Electric Stimulation Therapy[MeSH]) OR (Electrodes, Implanted[MeSH]) OR "10 kHz" OR "10 kHz" OR "1000 Hertz" OR "1000 Hz" OR "10-kHz high-frequenc*" OR "500 Hertz" OR "500 Hz" OR (HF stimulat*[tw]) OR (HF10 stimulat*[tw]) OR (high frequenc* stimulat*[tw]) OR (high-frequenc* peripheral[tw]) OR (high-frequenc* block*[tw]) OR (high-frequenc* waveform*[tw]) OR (high-frequenc* waveforms[tw]) OR (high-frequency paraesthesia-free[tw]) OR (high-frequency paresthesia-free[tw]) OR (kilohertz stimulat*[tw]) OR (kilohertz frequency[tw]) OR (kilohertz frequencies[tw]) OR (maximum voltage stimulat*[tw]) OR (maximal voltage stimulat*[tw]) OR (stimulat* electrode[tw]) OR (stimulat* electrodes[tw]) OR (burst[tw]) OR (“HF10 SCS”[tw]) OR (“HF10 Therap*”“[tw]) OR (HFSCS[tw]) OR (“high-frequency SCS”“[tw]) OR (high-frequency therap*[tw]) OR (kilohertz alternating current[tw]) OR (kilohertz alternating currents[tw]) OR (kilohertz electrical nerve block*[tw]) OR (neuromodulations[tw]) OR (neuromodulation[tw]) OR (neuro-modulation[tw]) OR (neuro-modulations[tw]) OR (paraesthesia-free[tw]) OR (paresthesia-free[tw])))))) AND ((((publisher[sb] NOT pubstatusnihms NOT pubstatuspmcsd NOT pmcbook) OR inprocess[sb] OR pubmednotmedline[sb] OR ((pubstatusnihms OR pubstatuspmcsd) AND publisher[sb])))) | 29 |

Biosys Previews

| History Name: | HFSCS and CRPS |
| --- | --- |
| Description: | High Frequency SCS Complex Regional Pain |

| **Set** | **Results** |  |
| --- | --- | --- |
| # 3 | [337](http://apps.webofknowledge.com.myaccess.library.utoronto.ca/summary.do?product=BIOSIS&doc=1&qid=7&SID=1DakWo9guTP2lK9xuSl&search_mode=CombineSearches&update_back2search_link_param=yes) | #2 AND #1  Indexes=BIOSIS Previews Timespan=All years |
| # 2 | [36,276](http://apps.webofknowledge.com.myaccess.library.utoronto.ca/summary.do?product=BIOSIS&doc=1&qid=4&SID=1DakWo9guTP2lK9xuSl&search_mode=AdvancedSearch&update_back2search_link_param=yes) | (TS=((Spinal Cord Stimulat*) OR (Electric Stimulation Therap*) OR (Electrode* NEAR/2 Implant*) OR "10 kHz" OR "10 kHz" OR "1000 Hertz" OR "1000 Hz" OR "10-kHz high-frequenc*" OR "500 Hertz" OR "500 Hz" OR (HF NEAR/2 stimulat*) OR (HF10 NEAR/2 stimulat*) OR (high frequenc* NEAR/2 stimulat*) OR (high-frequenc* NEAR/2 peripheral) OR (high-frequenc* NEAR/2 block*) OR (high-frequenc* NEAR/2 waveform*) OR (high-frequenc* NEAR/2 paraesthesia-free) OR (high-frequenc* NEAR/2 paresthesia-free) OR (kilohertz NEAR/2 stimulat*) OR (kilohertz NEAR/2 frequenc*) OR (kilo-hertz NEAR/2 frequenc*) OR (maximum voltage NEAR/2 stimulat*) OR (maximal voltage NEAR/2 stimulat*) OR (stimulat* NEAR/2 electrode*) OR (burst) OR (“HF10 SCS”) OR (“HF10 Therap*”) OR (HFSCS) OR (“high-frequency SCS”) OR (high-frequency NEAR/2 therap*) OR (kilohertz alternating current*) OR (kilohertz electrical NEAR/2 nerve block*) OR (neuromodulations) OR (neuromodulation) OR (neuro-modulation) OR (neuro-modulations) OR (paraesthesia-free) OR (paresthesia-free))) *AND* **TAXA NOTES:** (Humans)  Indexes=BIOSIS Previews Timespan=All years |
| # 1 | [5,325](http://apps.webofknowledge.com.myaccess.library.utoronto.ca/summary.do?product=BIOSIS&doc=1&qid=1&SID=1DakWo9guTP2lK9xuSl&search_mode=AdvancedSearch&update_back2search_link_param=yes) | (TS=((Complex Regional Pain Syndromee*) OR (Causalgi*) OR (Reflex Sympathetic Dystroph*) OR (CRPS) OR ("CRPS 1") OR ("CRPS 2") OR ("CRPS type 1") OR ("CRPS type 2") OR (algodystroph*) OR (causalgia) OR (causalgias) OR (Complex Regional Pain Syndrome*) OR (Morbus Sudeck) OR (neuralgic shoulder amyotroph*) OR (posttraumatic dystroph*) OR (post-traumatic dystroph*) OR (posttraumatic osteoporoses) OR (posttraumatic osteoporosis) OR (post-traumatic osteoporoses) OR (post-traumatic osteoporosis) OR (Reflex Sympathetic) OR (Reflex Sympathetic Dystrophy*) OR (shoulder arm syndrome*) OR (shoulder hand syndrome*) OR (sudeck* NEAR/2 atroph*) OR (sympathetic dystroph*) OR (sympathetic dystroph* syndrome*) OR (sympathetic reflex dystroph*) OR (Sudeck PH))) *AND* **TAXA NOTES:** (Humans)  Indexes=BIOSIS Previews Timespan=All years |

Scopus

**302 document results**

( ( TITLE-ABS-KEY ( ( ( spinal  AND cord  AND stimulat* )  OR  ( electric  AND stimulation  AND therap* )  OR  ( electrode*  W/2  implant* )  OR  "10 kHz"  OR  "10 kHz"  OR  "1000 Hertz"  OR  "1000 Hz"  OR  "10-kHz high-frequenc*"  OR  "500 Hertz"  OR  "500 Hz"  OR  ( hf  W/2  stimulat* )  OR  ( hf10  W/2  stimulat* )  OR  ( high  AND frequenc*  W/2  stimulat* )  OR  ( high-frequenc*  W/2  peripheral )  OR  ( high-frequenc*  W/2  block* )  OR  ( high-frequenc*  W/2  waveform* )  OR  ( high-frequenc*  W/2  paraesthesia-free )  OR  ( high-frequenc*  W/2  paresthesia-free )  OR  ( kilohertz  W/2  stimulat* )  OR  ( kilohertz  W/2  frequenc* )  OR  ( kilo-hertz  W/2  frequenc* )  OR  ( maximum  AND voltage  W/2  stimulat* )  OR  ( maximal  AND voltage  W/2  stimulat* )  OR  ( stimulat*  W/2  electrode* )  OR  ( burst )  OR  ( "HF10 SCS" )  OR  ( "HF10 Therap*" )  OR  ( hfscs )  OR  ( "high-frequency SCS" )  OR  ( high-frequency  W/2  therap* )  OR  ( kilohertz  AND alternating  AND current* )  OR  ( kilohertz  AND electrical  W/2  nerve  AND block* )  OR  ( neuromodulat* )  OR  ( neuro-modulat* )  OR  ( paraesthesia-free )  OR  ( paresthesia-free ) ) ) )  AND  ( TITLE-ABS-KEY ( ( ( complex  AND regional  AND pain  AND syndromee* )  OR  ( causalgi* )  OR  ( reflex  AND sympathetic  AND dystroph* )  OR  ( crps )  OR  ( "CRPS 1" )  OR  ( "CRPS 2" )  OR  ( "CRPS type 1" )  OR  ( "CRPS type 2" )  OR  ( algodystroph* )  OR  ( causalgia )  OR  ( causalgias )  OR  ( complex  AND regional  AND pain  AND syndrome* )  OR  ( morbus  AND sudeck )  OR  ( neuralgic  AND shoulder  AND amyotroph* )  OR  ( posttraumatic  AND dystroph* )  OR  ( post-traumatic  AND dystroph* )  OR  ( posttraumatic  AND osteoporoses )  OR  ( posttraumatic  AND osteoporosis )  OR  ( post-traumatic  AND osteoporoses )  OR  ( post-traumatic  AND osteoporosis )  OR  ( reflex  AND sympathetic )  OR  ( reflex  AND sympathetic  AND dystrophy* )  OR  ( shoulder  AND arm  AND syndrome* )  OR  ( shoulder  AND hand  AND syndrome* )  OR  ( sudeck*  W/2  atroph* )  OR  ( sympathetic  AND dystroph* )  OR  ( sympathetic  AND dystroph*  AND syndrome* )  OR  ( sympathetic  AND reflex  AND dystroph* )  OR  ( sudeck  AND ph ) ) ) ) )  AND NOT  ( PMID ( ( 0*  OR  1  OR  1*  OR  2*  OR  3*  OR  4*  OR  5*  OR  6*  OR  7*  OR  8*  OR  9* ) ) )  AND  ( EXCLUDE ( EXACTKEYWORD ,  "Nonhuman" )  OR  EXCLUDE ( EXACTKEYWORD ,  "Animal Experiment" )  OR  EXCLUDE ( EXACTKEYWORD ,  "Cat" )  OR  EXCLUDE ( EXACTKEYWORD ,  "Rat" )  OR  EXCLUDE ( EXACTKEYWORD ,  "Animal Model" ) )  AND  ( EXCLUDE ( DOCTYPE ,  "ch" ) )

ClinicalTrials.Gov

**15 Studies found for:**

**frequency | "Complex Regional Pain Syndrome" OR CRPS OR "Reflex Sympathetic Dystrophy"**

<https://clinicaltrials.gov/ct2/results?cond=%22Complex+Regional+Pain+Syndrome%22+OR+CRPS+OR+%22Reflex+Sympathetic+Dystrophy%22&term=frequency&cntry1=&state1=&Search=Search>

| **Row** | **Status** | **Study Title** | **Conditions** | **Interventions** |
| --- | --- | --- | --- | --- |
| 1 | Recruiting | [A Study to Confirm the Safety of High Frequency DRG Stimulator in Patients With Chronic Lower Limb Pain](https://clinicaltrials.gov/ct2/show/NCT03285113?term=frequency&cond=%22Complex+Regional+Pain+Syndrome%22+OR+CRPS+OR+%22Reflex+Sympathetic+Dystrophy%22&draw=1&rank=1) | Failed Back Surgery Syndrome;   Complex Regional Pain Syndrome (CRPS) | Device: GiMer Medical MN 1000 External Stimulator |
| 2 | Completed [Has Results](https://clinicaltrials.gov/ct2/show/results/NCT02265848?term=frequency&cond=%22Complex+Regional+Pain+Syndrome%22+OR+CRPS+OR+%22Reflex+Sympathetic+Dystrophy%22&draw=1&rank=2) | [High Frequency Stimulation Trials in Patients With Precision Spinal Cord Stimulator System](https://clinicaltrials.gov/ct2/show/NCT02265848?term=frequency&cond=%22Complex+Regional+Pain+Syndrome%22+OR+CRPS+OR+%22Reflex+Sympathetic+Dystrophy%22&draw=1&rank=2) | Chronic Pain;   Low Back Pain;   Radiculopathy;   Complex Regional Pain Syndrome (CRPS) | Other: High frequency stimulation;   Other: Low frequency stimulation |
| 3 | Unknown ^†^ | [Effects of Repetitive Electric Sensory Stimulation (RSS) as Intervention in Complex-regional-pain-syndrome Type I (CRPS)](https://clinicaltrials.gov/ct2/show/NCT01915329?term=frequency&cond=%22Complex+Regional+Pain+Syndrome%22+OR+CRPS+OR+%22Reflex+Sympathetic+Dystrophy%22&draw=1&rank=3) | Complex Regional Pain Syndrome Type I of the Upper Limb | Device: RSS (repetitive sensory stimulation);   Device: SHAM-RSS |
| 4 | Recruiting | [TMS for Complex Regional Pain Syndrome](https://clinicaltrials.gov/ct2/show/NCT03137472?term=frequency&cond=%22Complex+Regional+Pain+Syndrome%22+OR+CRPS+OR+%22Reflex+Sympathetic+Dystrophy%22&draw=1&rank=4) | Complex Regional Pain Syndromes | Device: Transcranial Magnetic Stimulation (TMS) |
| 5 | Completed | [Usefulness of C-reactive Protein Testing in Acute Cough](https://clinicaltrials.gov/ct2/show/NCT01794819?term=frequency&cond=%22Complex+Regional+Pain+Syndrome%22+OR+CRPS+OR+%22Reflex+Sympathetic+Dystrophy%22&draw=1&rank=5) | Respiratory Tract Infections | Device: C-reactive protein test |
| 6 | Completed | [C Reactive Protein (CRP) Intervention to Reduce Inappropriate Antibiotic Prescriptions in the Primary Healthcare Setting](https://clinicaltrials.gov/ct2/show/NCT01918579?term=frequency&cond=%22Complex+Regional+Pain+Syndrome%22+OR+CRPS+OR+%22Reflex+Sympathetic+Dystrophy%22&draw=1&rank=6) | Acute Respiratory Infections | Procedure: Patients will be tested by rapid POC CRP test |
| 7 | Completed | [Association of Transcutaneous Electrical Nerve Stimulation and Hypnosis](https://clinicaltrials.gov/ct2/show/NCT01944150?term=frequency&cond=%22Complex+Regional+Pain+Syndrome%22+OR+CRPS+OR+%22Reflex+Sympathetic+Dystrophy%22&draw=1&rank=7) | Limbs Arthrosis;   Non Arthrosic Limbs Arthralgia;   Chronic Lomboradiculalgia;   Chronic Back Pain;   Cervical Radiculopathy;   Post-herpetic Neuralgia;   Post-surgical Peripheral Neuropathic Pain;   Post Trauma Neuropathic Pain;   Complex Regional Pain Syndrome Type I or II;   Tendinopathy | Behavioral: Transcutaneous electrical nerve stimulation;   Behavioral: Transcutaneous electrical nerve stimulation and hypnosis |
| 8 | Unknown ^†^ | [Procalcitonin as a Marker of Infection in Cancer Patients](https://clinicaltrials.gov/ct2/show/NCT01227109?term=frequency&cond=%22Complex+Regional+Pain+Syndrome%22+OR+CRPS+OR+%22Reflex+Sympathetic+Dystrophy%22&draw=1&rank=8) | Infections;   Cancer;   Procalcitonin;   C Reactive Protein |  |
| 9 | Completed | [Novel Treatment Option for Neuropathic Pain](https://clinicaltrials.gov/ct2/show/NCT02490436?term=frequency&cond=%22Complex+Regional+Pain+Syndrome%22+OR+CRPS+OR+%22Reflex+Sympathetic+Dystrophy%22&draw=1&rank=9) | Neuralgia;   Complex Regional Pain Syndromes | Drug: Cetuximab;   Drug: Placebo |
| 10 | Completed | [Postoperative Complications After Appendectomy](https://clinicaltrials.gov/ct2/show/NCT03119740?term=frequency&cond=%22Complex+Regional+Pain+Syndrome%22+OR+CRPS+OR+%22Reflex+Sympathetic+Dystrophy%22&draw=1&rank=10) | Appendicitis |  |
| 11 | Completed | [Effects of Romantic Affection on Blood Chemistry and Immune Parameters](https://clinicaltrials.gov/ct2/show/NCT00482404?term=frequency&cond=%22Complex+Regional+Pain+Syndrome%22+OR+CRPS+OR+%22Reflex+Sympathetic+Dystrophy%22&draw=1&rank=11) | Stress;   Hypercholesterolemia | Behavioral: Romantic kissing |
| 12 | Completed [Has Results](https://clinicaltrials.gov/ct2/show/results/NCT02417376?term=frequency&cond=%22Complex+Regional+Pain+Syndrome%22+OR+CRPS+OR+%22Reflex+Sympathetic+Dystrophy%22&draw=1&rank=12) | [Changes in the Risk Factors of Coronary Heart Disease Observed After Scaling and Root Planing](https://clinicaltrials.gov/ct2/show/NCT02417376?term=frequency&cond=%22Complex+Regional+Pain+Syndrome%22+OR+CRPS+OR+%22Reflex+Sympathetic+Dystrophy%22&draw=1&rank=12) | Coronary Heart Disease;   Periodontitis | Device: piezoelectric ultrasonic scaler (frequency of 28-36 KHz);   Device: Gracey curettes |
| 13 | Recruiting | [The Impact of C-reactive Protein Testing](https://clinicaltrials.gov/ct2/show/NCT02758821?term=frequency&cond=%22Complex+Regional+Pain+Syndrome%22+OR+CRPS+OR+%22Reflex+Sympathetic+Dystrophy%22&draw=1&rank=13) | Fever | Other: No CRP will be measured onsite;   Other: CRP cut-off of 20mg/L.;   Other: CRP cut-off of 40mg/L. |
| 14 | Completed | [Positron Emission Tomography to Measure Pain and Pain Control](https://clinicaltrials.gov/ct2/show/NCT00001307?term=frequency&cond=%22Complex+Regional+Pain+Syndrome%22+OR+CRPS+OR+%22Reflex+Sympathetic+Dystrophy%22&draw=1&rank=14) | Healthy;   Hyperalgesia;   Pain;   Peripheral Nervous System Disease | Drug: Oxygen-15 Water;   Drug: Capsaicin |
| 15 | Completed | [Pilot Trial of Statin Use in Burn Patients](https://clinicaltrials.gov/ct2/show/NCT00978419?term=frequency&cond=%22Complex+Regional+Pain+Syndrome%22+OR+CRPS+OR+%22Reflex+Sympathetic+Dystrophy%22&draw=1&rank=15) | Burns | Drug: Rosuvastatin;   Drug: Placebo |

Potential Google Scholar String:

<https://scholar.google.ca/scholar?q=%28%28%22complex+regional+pain+syndrome%22+OR+%22reflex+sympathetic+dystrophy%22+OR+%22failed+back+syndrome%22%29+AND+%28%22high+frequency%22%29%29+AND+%28human+%7C+humans+%7C+patient+%7C+patients%29&btnG=&hl=en&as_sdt=1%2C5>

(("complex regional pain syndrome" OR "reflex sympathetic dystrophy" OR "failed back syndrome") AND ("high frequency")) AND (human | humans | patient | patients)

AHRQ – Health Technology Assessment & EPC

<https://www.ahrq.gov/research/findings/evidence-based-reports/search.html> 0 found

<https://www.ahrq.gov/research/findings/final-reports/index.html> 0 found

<https://www.ahrq.gov/research/findings/ta/index.html> 0 found

NIHR Health Technology Assessment Program

<https://www.nihr.ac.uk/research-and-impact/research/nihr-dissemination-centre.htm>

<https://discover.dc.nihr.ac.uk/portal/home>

0 found
