# Supplementary material for: A scoping review of novel spinal cord stimulation modes for complex regional pain syndrome
Source: Can J Pain. 2019 Mar 5;3(1):33–48. doi: 10.1080/24740527.2019.1574536 (PMC8730659; doi:10.1080/24740527.2019.1574536)
Supplement: Supplemental Material [file UCJP_A_1574536_SM9227.zip › Appendix 3 CJP assessment of risk of bias.docx]

**Appendix 3.**

| **Criterion** | **Judgement** | **Study 1**  **Al-Kaisy et al. 2015** | **Study 2**  **Reddy et al. 2016** | **Study 3**  **Wille et al. 2017** |
| --- | --- | --- | --- | --- |
| 1. **Is the hypothesis/aim/objective of the study clearly state?** | Yes/Unclear/ No | Yes | Yes | Yes |
| 1. **Are the characteristics of the participants included in the study described?** | Yes/Partially reported/No | Yes | Yes | Yes |
| 1. **Were the cases collected in more than one centre?** | Yes/Unclear/No | No | No | No |
| 1. **Are the eligibility criteria (i.e. inclusion and exclusion criteria) for entry into the study clearly stated?** | Yes/Partially reported/No | No | No | Yes |
| 1. **Were participants recruited consecutively?** | Yes/Unclear/No | Unclear | Yes | Yes |
| 1. **Did participants enter the study at a similar point in the disease?** | Yes/Unclear/No | Unclear | No | Unclear |
| 1. **Was the intervention of interest clearly described?** | Yes/Partially reported/No | Yes | Yes | Yes |
| 1. **Were additional interventions (co-interventions) reported in the study?** | Yes/Unclear/No | No | No | No |
| 1. **Are the outcome measures established a priori?** | Yes/Partially reported/No | No^*^ | Yes | No^*^ |
| 1. **Were the relevant outcomes measured with appropriate objective and/or subjective methods?** | Yes/Unclear/No | Yes | Yes | Yes |
| 1. **Were the relevant outcomes measured before and after the intervention?** | Yes/Unclear/No | Yes | Yes | Yes |
| 1. **Were the statistical tests used to assess the relevant outcomes appropriate?** | Yes/Unclear/No | Yes | Yes | Yes |
| 1. **Was the length of follow-up reported?** | Yes/Unclear/No | Yes | Yes | Yes |
| 1. **Was the loss to follow-up reported?** | Yes/Unclear/No | Yes | Yes | Yes |
| 1. **Does the study provide estimates of the random variability in the data analysis of relevant outcomes?** | Yes/Unclear/ Partially reported/No | Partially reported | Yes | Yes |
| 1. **Are the adverse events related with the intervention reported?** | Yes/Partially reported/No | Yes | Yes | No |
| 1. **Are the conclusions of the study supported by results?** | Yes/Partially reported/No | Yes | Yes | Yes |
| 1. **Are both competing interests and sources of support for the study reported?** | Yes/Partially reported/No | Partially reported | Partially reported | Partially reported |
| **Total number of “Yes” responses** |  | 10 | 13 | 12 |

^*^Retrospectively decided
